# Invariant Molecular Representations for Heterogeneous Catalysis

**DOI:** 10.1021/acs.jcim.3c00594

**Published:** 2024-01-10

**Authors:** Jawad Chowdhury, Charles Fricke, Olajide Bamidele, Mubarak Bello, Wenqiang Yang, Andreas Heyden, Gabriel Terejanu

**Affiliations:** † Department of Computer Science, University of North Carolina at Charlotte, Charlotte, North Carolina 28223, United States; ‡ Department of Chemical Engineering, University of South Carolina, Columbia, South Carolina 29208, United States

## Abstract

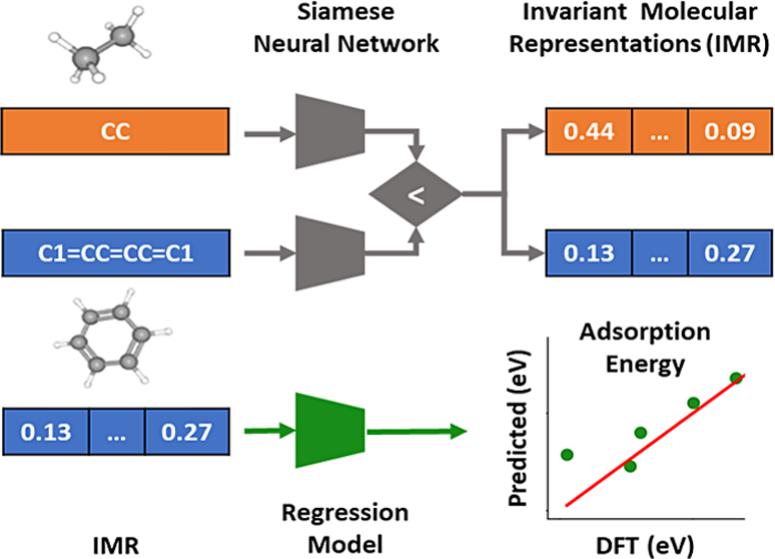

Catalyst screening is a critical step in the discovery and development of heterogeneous catalysts, which are vital for a wide range of chemical processes. In recent years, computational catalyst screening, primarily through density functional theory (DFT), has gained significant attention as a method for identifying promising catalysts. However, the computation of adsorption energies for all likely chemical intermediates present in complex surface chemistries is computationally intensive and costly due to the expensive nature of these calculations and the intrinsic idiosyncrasies of the methods or data sets used. This study introduces a novel machine learning (ML) method to learn adsorption energies from multiple DFT functionals by using invariant molecular representations (IMRs). To do this, we first extract molecular fingerprints for the reaction intermediates and later use a Siamese-neural-network-based training strategy to learn invariant molecular representations or the IMR across all available functionals. Our Siamese network-based representations demonstrate superior performance in predicting adsorption energies compared with other molecular representations. Notably, when considering mean absolute values of adsorption energies as 0.43 eV (PBE-D3), 0.46 eV (BEEF-vdW), 0.81 eV (RPBE), and 0.37 eV (scan+rVV10), our IMR method has achieved the lowest mean absolute errors (MAEs) of 0.18 0.10, 0.16, and 0.18 eV, respectively. These results emphasize the superior predictive capacity of our Siamese network-based representations. The empirical findings in this study illuminate the efficacy, robustness, and dependability of our proposed ML paradigm in predicting adsorption energies, specifically for propane dehydrogenation on a platinum catalyst surface.

## Introduction

The development of efficient and cost-effective catalysts is of great importance in the chemical industry, as catalysts play a crucial role in a wide range of chemical reactions to increase the efficiency and selectivity of these processes.^1^ In recent years, computational catalyst screening has gained significant attention as a method for identifying promising catalysts, as it allows for the rapid and efficient evaluation of large numbers of potential catalysts.^2,3^ Computational catalysis involves the use of density functional theory (DFT) to compute the energy of the adsorption and transition states of various elementary chemical processes occurring on a catalyst surface.^4^ Macroscopic observables similar to those measured experimentally are then computed using transition state theory and a microkinetic model (MKM), taking the DFT-computed adsorption and transition state energies as inputs.^5^ However, the computation of adsorption and transition state energies using DFT can be both cost and time-intensive; hence, for most reaction systems of importance involving many intermediates or adsorbate species, the computation of adsorption energy is an arduous and expensive task.^6^ DFT employs exchange–correlation functional approximations to account for the complex many-body electron–electron interaction terms in the time-independent Schrodinger equation (TISE) that describes the stationary state of a quantum mechanical system. Therefore, many DFT functionals exist, with each differing in the level of theory and inherent approximations. Hence, DFT-derived adsorption energies and consequently macroscopic observations depend on the choice of functionals. DFT functionals commonly used within the catalysis community include PBE-D3, RPBE, BEEF-vdW, and SCAN + rVV10. Adsorption energy, which quantifies a molecule’s binding strength to a catalyst’s surface, is directly computable via DFT. While not directly indicative of catalysis speed, it provides insights into molecule stability on the catalyst, influencing potential reaction pathways. For an in-depth exploration of the fundamental principles of heterogeneous catalysis, readers are referred to the seminal work, “Fundamental Concepts in Heterogeneous Catalysis” by Nørskov et al.^4^ Nevertheless, the computation of adsorption energies for a large number of intermediates likely present and kinetically relevant in a chemical process can be computationally costly and even prohibitive due to the expensive nature of these calculations.^7^

To address the high computational cost of calculating adsorption and transition state energies for various active site models, linear scaling relations^7−9^ have been developed for surface intermediates and transition states that use few computable descriptors to generate volcano curves on catalyst activity.^10^ Nevertheless, the effectiveness of these relations for more complex chemistries remains uncertain. Additionally, the process of selecting descriptors for these calculations often involves a trial-and-error approach. In contrast, a more systematic approach was proposed in a previous study,^11^ which used principal component analysis (PCA)^12,13^ to identify the optimal minimal set of descriptors for the calculations, outperforming conventional descriptor selection methods. To overcome the computational challenge and to predict properties of the chemical entities using machine learning,^14,15^ the commonly employed approach often takes place in two major steps. First, a suitable and effective descriptor is selected in the initial step, followed by the use of machine learning techniques to predict these chemical properties in the second step. Machine learning models can be trained using data obtained from DFT calculations and can subsequently be used to predict adsorption energies for a broad range of intermediates and catalysts.^16,17^

In addition to the computational cost, predicting the adsorption energy of reaction intermediates and learning from multiple functionals can be a daunting task due to the intricate nature of the interactions between the intermediates and the catalyst surface, as highlighted in previous literature.^18,19^ Machine learning models typically perform well when applied to the same or similar domains or functionals on which they were trained, but their performance can be severely compromised when extrapolated to different functionals.^20^ The efficacy of the prediction results is largely dependent on the selection of the functionals used in the calculations and their inherent idiosyncrasies. The utilization of different functionals can result in varying predictions and models, making it challenging to determine the most accurate functional for a particular system. Moreover, the accuracy of the predictions can be further hampered by the quality of the training data, which are often obtained through experiments or DFT calculations. This limited set of training data, combined with the peculiarities of the functionals, results in a high level of uncertainty in the predictions, which poses a significant challenge to accurately predicting the adsorption energy of reaction intermediates from different functionals.^21,22^ Therefore, despite their capability to capture complex interactions between intermediates and the surface, existing machine learning strategies are hindered by the differences in functionals and their lack of generalization capability.

Our study proposes a novel approach to address the limitations of current methods for predicting the adsorption energy of reaction intermediates across different functionals. Our approach demonstrates that multiple functionals can benefit learning rather than impede it and effectively overcome the difficulties associated with the unique characteristics of individual functionals. The proposed method involves capturing the relative energy differences between pairs of intermediates calculated within the same functional and training the model across all of the different functionals. This strategy results in a robust, reliable, and generalizable molecular representation across different functionals, representing a significant advancement in this direction.

To achieve this, our implementation involves the extraction of molecular fingerprints and the training of Siamese neural networks on these fingerprints across different functionals with the aim of learning invariant molecular representations (IMRs) for catalysis. Molecular fingerprints provide numerical representations of molecules that encode their chemical properties and can serve as inputs for machine learning models. Several fingerprint generation schemes have been proposed in the literature, including the Coulomb matrix^23^ and bag-of-bonds^24^ approaches that use distance measures between the atomic coordinates of a species, as well as atom-centered radial or angular symmetry functions.^25−28^ Non-coordinate-based fingerprints that consider features of a molecule that can be extracted from its chemical formula or SMILES notation have also been developed.^29−32^ SMILES, which stands for the Simplified Molecular Input Line Entry System, is a compact and intuitive notation system for representing the molecular structure of a compound. SMILES notation can be used to generate fingerprints that capture the structural and chemical features of a molecule. Fingerprints can also be generated from the molecular graph structure, treating atoms and bonds as nodes and edges, respectively.^33,34^ They may also be tailored to correspond to a specific property to be learned through backpropagation or other techniques. Interestingly, SMILES or graph-based fingerprints have a desirable property over coordinate-based descriptors in that any DFT or semiempirical methods need only to be applied to the training data, unlike coordinate-based methods require reliable atomic coordinates even for species in the prediction set, potentially necessitating expensive calculations. Kernel-based models, such as kernel ridge regression,^35^ and neural network-based models, such as graph convolution,^33,34^ recurrent neural networks,^36^ and three-dimensional (3D) convolutional neural networks,^37^ have been widely used in this context. Some studies have also employed additive atomic contributions through atomic subnetworks.^14,25^

In this study, we have applied a novel approach by utilizing Siamese neural networks,^38,39^ a type of neural network architecture well-suited for comparing pairs of input data and determining their similarity, to learn from the relative comparison of molecular pairs. The aim of our study is to generate molecular representations that capture inherent similarities and dissimilarities between pairs of molecules, with the intention to enhance the predictive capability of adsorption energies for reaction intermediates by leveraging additional functionals. “Functionals” in the context of our study refer to exchange–correlation functionals within DFT. The exchange–correlation functional approximation makes Kohn–Sham DFT a practical method for predicting the energy of a system. And when we refer to “invariant molecular representations”, we are indicating representations that are robust to the variations introduced by these different DFT functionals rather than the traditional invariance associated with rotation, translation, and exchange of atoms often seen in molecular descriptor engineering. This approach allows us to capture the underlying chemistry of the system in a manner that is insensitive to the choice of functional while being informed by the specific system the model is trained on. To validate our approach, we applied it to the prediction of adsorption energies for propane dehydrogenation on a platinum catalyst surface and found it to be significantly superior and reliable in its predictive performance across different experimental settings. These results demonstrate the potential of our novel approach in aiding the design and optimization of catalysts for chemical reactions.

## Methodology

The purpose of this section is to present a comprehensive overview of our proposed approach for predicting the adsorption energies of reaction intermediates on the catalyst surface. While our experimental case studies focused on the prediction of adsorption energies for propane dehydrogenation on a platinum catalyst surface using three different types of constant-size molecular fingerprints to generate invariant representations, it is important to note that our proposed method is not limited to these specific choices of data. The proposed method is more generalizable and can be applied to other types of molecular descriptors, as well.

The section is structured as follows: First, we provide an overview of the data collection and preparation process. Next, we will discuss the species descriptors or fingerprints we have employed, the structure of our proposed model, and the training strategies used to generate molecular representations. Finally, we outline the process for the predictive modeling of adsorption energies from the IMR or invariant molecular representations generated.

### Data Set—Data Collection and Preparation

It is well-established that adsorption energies of molecular species can vary significantly depending on the metal surface being examined.^4^ For the purpose of our experiments, we utilized data on propane dehydrogenation on a platinum surface model.^40^ The calculations for the four DFT functionals, namely, PBE-D3,^41,42^ RPBE,^43^ BEEF-vdW,^22^ and SCAN + rVV10,^44^ were performed using the Vienna Ab initio Simulation Package (VASP) version 5.4.4.^45−47^ Additionally, data for training with random BEEF-vdW ensembles was generated using an ensemble of 2000 non-self-consistent field (NSCF) energies. The NSCF energies are computed using various possible exchange–correlation functionals (within the generalized gradient approximation) while using the RPBE electron densities that are self-consistent only for the RPBE functional. This is the conventional procedure for BEEF-vdW calculations. The data preparation step with training strategies employed in our proposed model will be further elaborated upon in later sections. Our data set consists of 46 intermediate species along with their SMILES notations. SMILES notation is a naming convention for chemical species with a set of rules allowing for a unique representation of each chemical species. For instance, in SMILES nomenclature, “C” represents a fully saturated and stable single-carbon molecule, i.e., CH_4_ (methane). Unstable molecules or intermediates are denoted within square brackets, such as [CH_3_], [CH_2_], etc. Double and triple bonds are represented as “=” and “#”, respectively, while branched species are indicated within parentheses “()”. The data set also includes the adsorption energies for the Pt(111) metal surface calculated using all four DFT functionals and the 2000 BEEF-vdW ensembles. Additional details on the DFT calculations can be found in the Supporting Information.

### Molecular Fingerprints

Molecular fingerprints can be broadly classified into two categories: 3D fingerprints (coordinate-based) and topological/2D fingerprints (non-coordinate-based). 3D fingerprints suffer from the limitation that they require computationally expensive methods, such as DFT, or other semiempirical techniques for their generation. In contrast, topological 2D fingerprints offer an alternative means for generating molecular fingerprints from SMILES notations that do not involve such computationally intensive processes. Notably, the molecular fingerprints employed in this study do not include any information regarding the catalyst, since all species are adsorbed on the same Pt(111) catalyst surface. We utilized three different noncoordinate-based techniques to obtain the fingerprints from SMILES notations of the molecular species. The following section provides a brief discussion of the three techniques.

#### Flat Molecular Fingerprints (24-Length)

**Figure 1:**
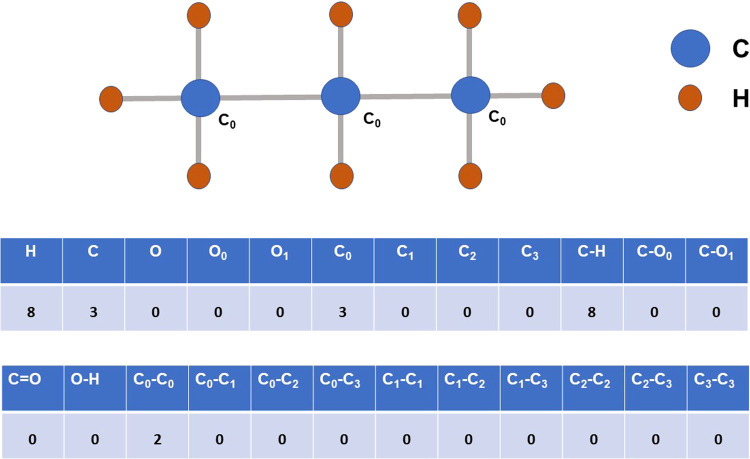
24-length flat molecular fingerprints for the species CH_3_CH_2_CH_3_. Here, *C*_0_ denotes carbon atoms with no free valence (saturated carbon), whereas *C*_1_, *C*_2_, and *C*_3_ denote carbon atoms with one, two, and three free valencies. This type of fingerprint contains information based on the number of saturated and unsaturated atoms and the number of bond counts between them.

Molecular fingerprints are often used to represent the molecular structure of a species. These fingerprints can be generated by using various methods, including counting the different bond types present around each atom in a molecular species. In this study, we have employed constant-sized flat molecular fingerprints,^48^ similar to the approach in reference study,^16^ based on the SMILES notation of each species. Specifically, we generated 24-length flat molecular fingerprints, which capture information about the number of different bond types present in the molecule. An example of the molecular fingerprints generated by using this method is shown in Figure 1. These fingerprints provide more information than a basic bond count scheme, as they also take into account the number of free valencies and more granular bond count information related to the free valencies. This enables a more comprehensive understanding of the structural features of the molecule.

#### chEMBL Fingerprints (768-Length)

In this study, we have adopted the pretrained chEMBL model^49^ as an alternative method for generating molecular fingerprints. The chEMBL model is an approximation to the generative recurrent networks for de novo drug design in a prior study,^50^ primarily intended to capture the syntax of molecular structure in terms of SMILES strings. The resulting learned pattern probabilities can be used for de novo SMILES generation, making these pretrained models widely employed in chemogenomics and de novo drug design. The chEMBL model employed in this study is a mask language model (MLM). An MLM is basically a neural network trained to predict missing words in a text, enabling it to learn the underlying structure and relationships among the words in the text. The chEMBL model was trained from scratch using 438,552 SMILES notations and generates 768-length molecular fingerprints for each species, yielding a rich representation of the structural features of the molecule. These fingerprints have a broad range of applications, including the prediction of chemical properties and the design of novel compounds.

#### Morgan Fingerprints (24-Length)

Building on our exploration of molecular fingerprints, we incorporated the Morgan fingerprints^51^ for their unique approach to capturing molecular features. Circular by design, Morgan fingerprints focus on the molecular environment of each atom within a specified radius. By iterating over concentric bonds around each atom, this methodology generates a descriptor that identifies not only the type of atom but also its distinct local environment. In our study, we converted the SMILES notation of each species into 24-length Morgan fingerprints by setting a radius of 2. This ensures that the fingerprint captures the molecular environment up to two bonds out from each atom (neighbors and neighbors of neighbors). This choice in the descriptor, combined with our other fingerprint methods, enhances our study’s depth, further enriching the comprehension of molecular structures in our data set.

### Molecular Representations

**Figure 2:**
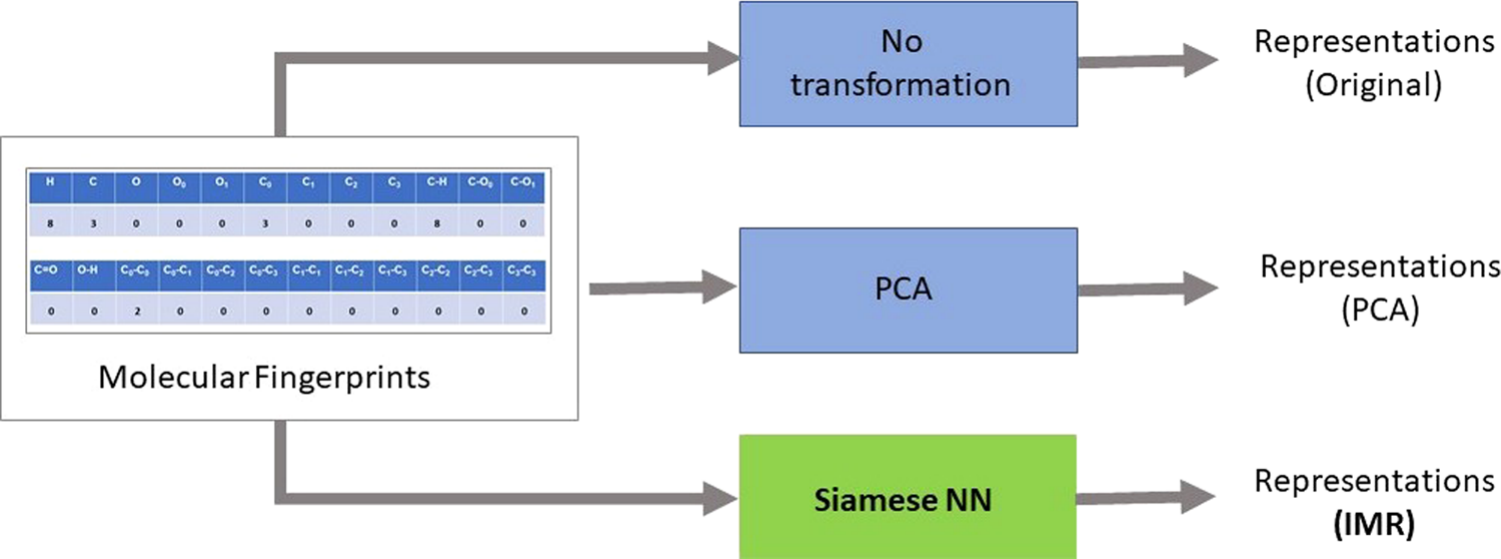
Three different methods were adopted to generate molecular representations from molecular fingerprints. Top: (Original) No transformation or modification is done on the original or raw fingerprints. Middle: (PCA) Molecular representations generated based on principal component analysis. Bottom: (IMR) Molecular representations generated using our trained Siamese neural network model.

We have examined three distinct methods for generating molecular representations from the fingerprints: the raw or original fingerprints (Original), PCA-based (PCA), and Siamese-based (IMR). The Original method involves using the raw fingerprints obtained from SMILES notations without any additional transformation. The second method employs principal component analysis (PCA)^12,13^ to transform the raw fingerprints into low-dimensional molecular representations. This approach is motivated by previous research^11^ that found PCA to be effective in obtaining descriptors over other conventional descriptors. For the PCA-based molecular representations, we have used the representation size that explains approximately 98% of the variance for 24-length flat molecular and Morgan fingerprints and 90% of the variance for chEMBL model-based fingerprints. Our third method, referred to as IMR, utilizes a trained Siamese neural network to generate invariant molecular representations from the raw fingerprints. This allows for a comparison of the performance of different representation generation methods and identification of those that are most effective for predictive modeling tasks. The process of generating these molecular representations from fingerprints is illustrated in Figure 2. Overall, our study enables the evaluation of the relative effectiveness of various approaches for generating molecular representations with potential applications in predictive modeling tasks. It is worth noting that the representations obtained in this study are essentially transformed fingerprints, which either aim to reduce the dimensionality of the fingerprints (PCA) or incorporate knowledge about the catalyst (IMR).

### Structure of the Proposed Model

The fundamental aim of our study is to develop robust molecular representations for reaction intermediates that can be generalized across multiple functionals. The Siamese neural network^39^ is selected for its ability to compare input data pairs and identify their similarities and differences. Our hypothesis is that training the network using all functionals in the training set for a given surface will enable it to generate invariant and informative representations of molecular species from raw fingerprints. These representations should be free from information specific to individual functionals but should be aware of the surface system, allowing the resulting model to be more generalizable.

The Siamese neural network comprises two identical subnetworks with identical weights and architecture, which are used to process and analyze the molecular fingerprints for each pair of molecular species across all different functionals in the training set. These subnetworks generate molecular representations that are then fed into a feedforward neural network. These subnetworks are intended to identify informative information from the raw fingerprints and provide an intermediate representation that the feedforward network can use to learn the relative energy differences. The overall Siamese network is trained to predict the relative difference in adsorption energies of the pair by using mean absolute errors (MAEs) as the cost function.

**Figure 3:**
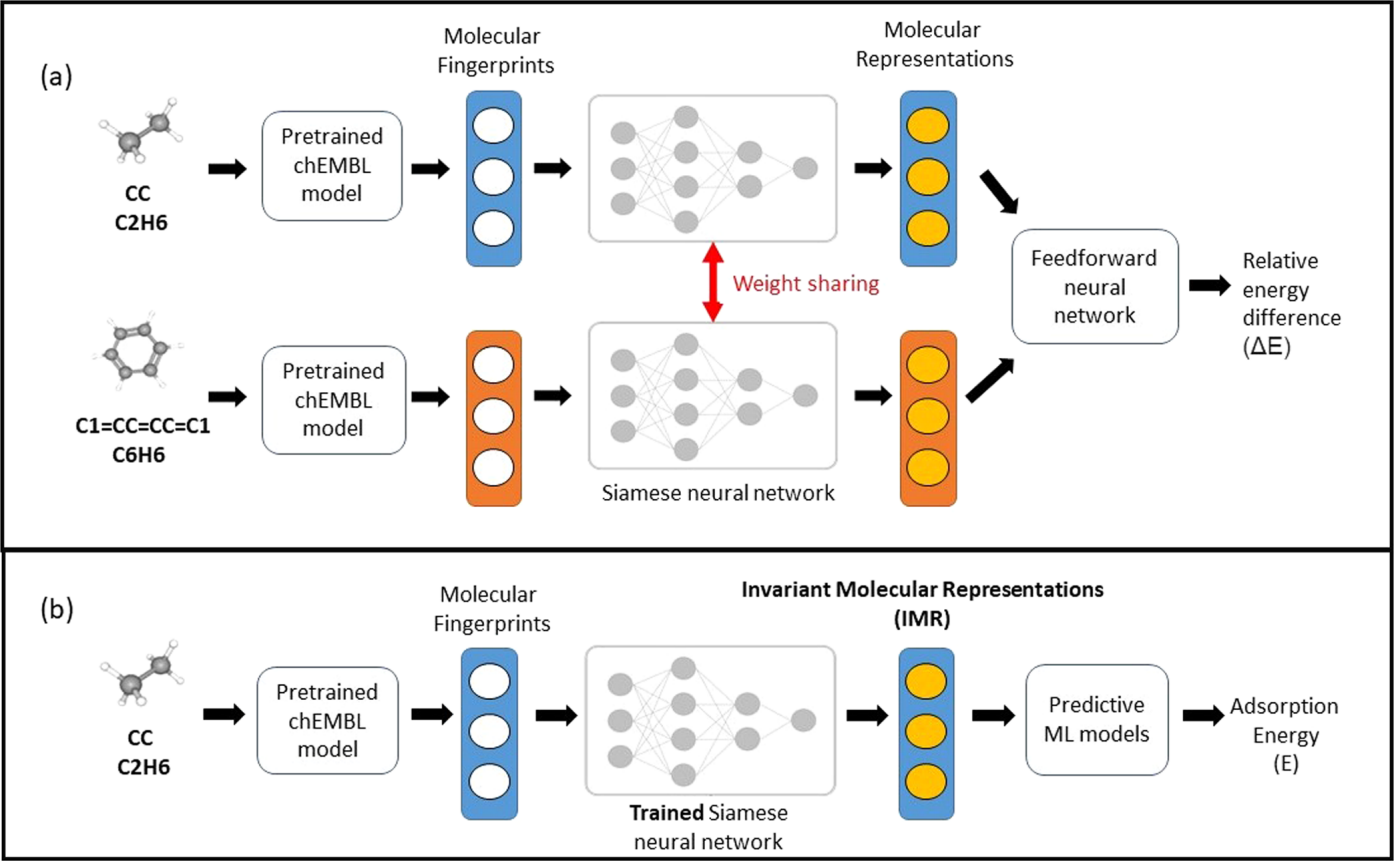
Two major steps of our proposed method/pipeline: (a) training the Siamese neural network to generate invariant molecular representations (IMR) across different functionals using relative energy differences between species and (b) predictive modeling of adsorption energies using IMR generated by the Siamese model trained in step (a).

Nonlinearity is incorporated through nonlinear activation functions^52−55^ used in the hidden layers of the Siamese networks. The subnetworks are randomly initialized,^56,57^ and their weights are updated during training to minimize the MAEs. Figure 3(a) illustrates the overall training process of our proposed network. To ensure a fair comparison, we used the same representation size for the Siamese network to generate representations (IMR) as the number of components used for the corresponding PCA-based molecular representations (PCA).

### Training Strategies Using the Proposed Model

The training of the Siamese neural network in this study involved three distinct strategies. The first two strategies, namely, (i) four functional model (FFM) training and (ii) BEEF-vdW ensemble model (BEM) training, made use of additional functional information to generate more meaningful and concise molecular representations for the predictive modeling tasks. The third training strategy, termed functional specific model (FSM) training, served as a validation check for the results obtained. Further details on these training strategies are presented in the corresponding experimental result sections.

### Predictive Modeling with the Proposed Model

In our study, we propose a predictive modeling mechanism for adsorption energies, as illustrated in Figure 3(b). The first step involves extracting molecular fingerprints based on atomic bond types and counts (flat fingerprints), utilizing the pretrained chEMBL model (chEMBL fingerprints) or using the Morgan fingerprints. Subsequently, the Siamese neural networks are trained using the training strategies and steps outlined in the previous section, as shown in Figure 3(a). The resulting trained subnetwork generates IMR for our training samples of molecular species. Finally, we utilize various machine learning algorithms, including ridge, elastic net, kernel ridge, and support vector regression, to train on the IMR generated by the trained subnetwork and conduct predictive analysis of the adsorption energies of corresponding molecular samples.

The motivation behind utilizing predictive modeling algorithms on top of the IMR, or in other words, the representations obtained from the trained Siamese network, can be explained as follows. First, with the Siamese network, we aim to learn molecular representations that are invariant across different functionals. We anticipate that these representations would capture the relationship of the fingerprints to the adsorption energies without relying on the functional but rather considering only the surface system and molecular structures. However, to evaluate the informativeness of IMR toward learning the adsorption energies, we employ functional-dependent predictive models as the second step. In our studies, we used four machine learning algorithms, namely, ridge, elastic net, kernel ridge, and support vector regression, to generate functional-dependent predictive models.

In summary, the input for the predictive ML models was the IMR or the invariant molecular representations of the species generated by our proposed model and training strategy from the raw fingerprints, and the outputs were the actual adsorption energies of those species. Later on, we evaluated the performance of the IMR in comparison to raw or the original fingerprints (Original) and PCA-based representations (PCA) on predictive analysis.

## Results and Discussion

In the following sections, we provide a detailed description of the simulation process and results for all of our case studies. Our observations on the results are also presented.

### Simulation

Given the inherent challenges posed by our data set size of 46 molecules, we meticulously adopted a robust approach. For each of our experimental case studies, we conducted 10 random trials, with each trial utilizing a different randomly chosen training and test set at a 2:1 ratio (i.e., approximately 67% of the data for training and 33% for testing), ensuring enhanced variability and consistency in the results.

For each trial, the training data were further subjected to 5-fold cross-validation, where the training set was divided into five subsets. Four of these subsets were used for ML model training for predicting the adsorption energies, while the fifth subset served as the validation set. This procedure was repeated 5 times, each time with a different subset of the validation set. The model parameters were optimized based on the performance across these validation sets.

We evaluated three different molecular representations in predicting the adsorption energies of various species based on the mean and standard deviations of the mean absolute errors (MAEs). To reinforce the reliability of our observations, we employed a *t*-test with α = 0.05 as the significance level. Furthermore, to safeguard against potential biases and to encompass a broad spectrum of analysis, for each experimental case, we employed four distinct machine learning algorithms: ridge regression (ridge), elastic net regression (elastic), support vector regression (svr), and kernel ridge regression (krr).

### Four Functional Model (FFM) Training

We first employed a training and testing strategy utilizing data from four distinct DFT functionals: PBE-D3, BEEF-vdW, RPBE, and SCAN + rVV10. We designed four experimental scenarios, each targeting the predictive performance for one of these functionals. For each case, the data from the test functional was reserved, and the Siamese network was trained using molecular species pairs from the remaining three functionals to predict their “relative energy difference”. Once trained, this network effectively translated molecular fingerprints into representations.

To assess these representations’ predictive capability for adsorption on the test functional, we first randomly partitioned its data into training and test sets for each trial. We then trained various ML models on the training data using the representations derived from our trained Siamese network. Subsequently, the performance of these ML models was evaluated on the held-out test samples from the test functional. The resulting mean absolute errors (MAEs) indicate the discrepancies between the predicted adsorption energies and the DFT-calculated ones.

To evaluate our method’s generalization capacity, we tested it using four ML models: ridge, elastic, krr, and svr. Each model was trained on three different representations (Original, PCA, and IMR). The Originals are unmodified fingerprints; PCA representations come from principal component analysis applied to these fingerprints, while IMR uses the Siamese network-trained representations.

By evaluating IMR’s performance on data excluded from the corresponding functional during Siamese model training, we could assess its capability to adapt to novel DFT functionals, showcasing our approach’s broad applicability.

#### Representations Generated from Flat Fingerprints

We initially examined the experimental results using an FFM training strategy and representations generated from flat molecular fingerprints, which are based on atomic bond types and counts in each molecular species (as mentioned in detail in the earlier section). Table 1 presents the corresponding outcomes, which display the test functional used in each experimental case (Test Functional), the machine learning algorithm employed for predictions of adsorption energies (ML Alg.), and the type of input representations used, namely, Original, PCA, and IMR. Our empirical findings indicate statistically significant improvements in the use of molecular representations learned by the Siamese network (IMR) compared to PCA-based representations (PCA) for three out of four functionals (BEEF-vdW, RPBE, and SCAN + rVV10). For PBE-D3, we also observed improvements when using IMR, although this was statistically significant in the case of the “ridge” and “elastic net” as the predictive algorithm.

**Table 1:** Evaluation of Three Molecular Representations (Original, PCA, and IMR) Using 24-Length Flat Molecular Fingerprints and the FFM Strategy^a^

test functional	ML Alg.	original	PCA	IMR
PBE-D3	ridge	0.31 ± 0.04	0.32 ± 0.04	**0.26 ± 0.05**
	elastic	0.33 ± 0.04	0.34 ± 0.05	**0.27 ± 0.06**
	krr	0.35 ± 0.05	0.33 ± 0.06	0.29 ± 0.03
	svr	0.31 ± 0.06	0.30 ± 0.06	0.29 ± 0.04
BEEF-vdW	ridge	0.31 ± 0.05	0.31 ± 0.05	**0.16 ± 0.03**
	elastic	0.32 ± 0.05	0.33 ± 0.04	**0.16 ± 0.02**
	krr	0.33 ± 0.04	0.34 ± 0.05	**0.15 ± 0.04**
	svr	0.31 ± 0.05	0.31 ± 0.06	**0.14 ± 0.04**
RPBE	ridge	0.31 ± 0.05	0.31 ± 0.05	**0.22 ± 0.06**
	elastic	0.33 ± 0.05	0.33 ± 0.05	**0.20 ± 0.05**
	krr	0.35 ± 0.05	0.36 ± 0.04	**0.22 ± 0.05**
	svr	0.32 ± 0.07	0.34 ± 0.07	**0.20 ± 0.04**
SCAN + rVV10	ridge	0.37 ± 0.05	0.38 ± 0.04	**0.25 ± 0.06**
	elastic	0.40 ± 0.04	0.39 ± 0.04	**0.24 ± 0.03**
	krr	0.42 ± 0.08	0.42 ± 0.07	**0.23 ± 0.05**
	svr	0.38 ± 0.08	0.39 ± 0.10	**0.24 ± 0.06**

a Displayed values are Mean Absolute Errors (MAEs) between predicted and DFT-calculated energies in electron volts (eV). Lower MAEs signify better performance. Bold values are statistically significant based on the *t*-test.

#### Representations Generated from Transfer Learning

Subsequently, we investigated the performance of the FFM training approach but this time with representations generated by using fingerprints from the chEMBL model. The results of the experimental cases are presented in Table 2.

**Table 2:** Evaluation of Three Molecular Representations (Original, PCA, and IMR) Using 768-Length Fingerprints from the chEMBL Model and FFM Strategy^a^

test functional	ML Alg.	original	PCA	IMR
PBE-D3	ridge	0.39 ± 0.06	0.37 ± 0.05	**0.24 ± 0.06**
	elastic	0.32 ± 0.07	0.32 ± 0.04	**0.23 ± 0.04**
	krr	0.27 ± 0.07	0.28 ± 0.05	**0.19 ± 0.07**
	svr	0.28 ± 0.06	0.28 ± 0.06	**0.18 ± 0.06**
BEEF-vdW	ridge	0.42 ± 0.07	0.36 ± 0.05	**0.14 ± 0.04**
	elastic	0.34 ± 0.05	0.33 ± 0.04	**0.14 ± 0.05**
	krr	0.34 ± 0.06	0.32 ± 0.05	**0.15 ± 0.07**
	svr	0.32 ± 0.05	0.34 ± 0.04	**0.14 ± 0.05**
RPBE	ridge	0.46 ± 0.08	0.39 ± 0.05	**0.18 ± 0.05**
	elastic	0.37 ± 0.05	0.38 ± 0.04	**0.19 ± 0.06**
	krr	0.44 ± 0.07	0.37 ± 0.04	**0.22 ± 0.06**
	svr	0.38 ± 0.05	0.39 ± 0.04	**0.20 ± 0.05**
SCAN + rVV10	ridge	0.44 ± 0.06	0.39 ± 0.05	**0.23 ± 0.06**
	elastic	0.39 ± 0.05	0.38 ± 0.03	**0.23 ± 0.06**
	krr	0.37 ± 0.04	0.39 ± 0.03	**0.22 ± 0.03**
	svr	0.35 ± 0.05	0.40 ± 0.05	**0.19 ± 0.03**

a The values presented are mean absolute errors (MAEs) between the predicted and DFT-calculated adsorption energies, measured in electron volts (eV). Smaller MAEs signify better performance. Bold values are statistically significant via *t*-test.

Notably, using representations generated from chEMBL fingerprints also demonstrated a clear trend of statistically significant improvements when adopting IMR compared to PCA representations for all four DFT functionals. However, if we consider the performance of the Original representations, the errors varied more widely among the different predictive machine learning algorithms (ridge, elastic, KRR, and SVR) compared to those obtained using the flat fingerprints. This indicates that in some instances, the machine learning models struggled to fit properly to the Original representations, as the feature dimension (*d* = 768) in these Original representations was much larger (*d*≫) compared to the number of samples/species (*n* = 46).

#### Representations Generated from Morgan Fingerprints

Moving on to our results derived from Morgan fingerprints, we applied the same FFM training strategy. The details of this analysis can be found in Table 3. Similar to our previous findings, the molecular representations processed by the Siamese network (IMR) stood out, demonstrating superior performance over PCA-based representations, particularly for the BEEF-vdW, RPBE, and SCAN + rVV10 functionals. As for PBE-D3, while there was an evident improvement with the use of IMR, it was statistically significant only in the case when we employed the elastic net’ as our regression algorithm.

**Table 3:** Performance Evaluation of Three Molecular Representations (Original, PCA, and IMR) Using 24-Length Morgan Fingerprints and the FFM Training Strategy^a^

test functional	ML Alg.	original	PCA	IMR
PBE-D3	ridge	0.34 ± 0.05	0.33 ± 0.05	0.34 ± 0.11
	elastic	0.32 ± 0.05	0.31 ± 0.05	**0.25 ± 0.04**
	krr	0.31 ± 0.05	0.33 ± 0.07	0.28 ± 0.05
	svr	0.31 ± 0.05	0.31 ± 0.05	0.28 ± 0.06
BEEF-vdW	ridge	0.34 ± 0.04	0.34 ± 0.04	**0.11 ± 0.04**
	elastic	0.33 ± 0.03	0.34 ± 0.04	**0.11 ± 0.04**
	krr	0.33 ± 0.04	0.34 ± 0.04	**0.10 ± 0.03**
	svr	0.33 ± 0.05	0.32 ± 0.05	**0.10 ± 0.03**
RPBE	ridge	0.37 ± 0.06	0.37 ± 0.06	**0.18 ± 0.03**
	elastic	0.37 ± 0.05	0.38 ± 0.05	**0.17 ± 0.03**
	krr	0.38 ± 0.05	0.39 ± 0.05	**0.17 ± 0.04**
	svr	0.38 ± 0.06	0.38 ± 0.06	**0.17 ± 0.04**
SCAN + rVV10	ridge	0.39 ± 0.05	0.39 ± 0.05	**0.22 ± 0.05**
	elastic	0.40 ± 0.03	0.40 ± 0.04	**0.20 ± 0.06**
	krr	0.39 ± 0.04	0.39 ± 0.03	**0.18 ± 0.03**
	svr	0.39 ± 0.05	0.39 ± 0.05	**0.19 ± 0.04**

a The values presented are mean absolute errors (MAEs) between the predicted and DFT-calculated adsorption energies in electron volts (eV). A smaller MAE value indicates a better performance. Values highlighted in bold are determined to be statistically significant via *t*-test.

### BEEF-vdW Ensemble Model (BEM) Training

Next, we adopted a similar approach to the FFM training, with a few key differences. To train our predictive machine learning models, namely, ridge, elastic, krr, and svr, we have utilized data from one of the four DFT functionals in each case focusing on learning the predictive performance on that specific functional. However, instead of using the remaining three DFT functionals to train our Siamese network (as we have seen in FFM training), we used the BEEF-vdW ensembles, and the Siamese network was trained using molecular species pairs from these BEEF-vdW ensembles to predict their “relative energy difference”.

The BEEF-vdW functional produces an ensemble of 2000 NSCF energies for each species. In every trial, we randomly select an ensemble of 50 BEEF-vdW functional energies from the available 2000. This allows us to treat each BEEF-vdW ensemble functional as distinct, leveraging them as different functionals—a novel approach in physical chemistry to our knowledge.

Upon training, the Siamese network adeptly converts molecular fingerprints into representations. To assess the predictive power of these representations for adsorption on a test functional, we partitioned its data into training and test sets for each trial. Different ML models are trained on the training set by using the representations generated from the trained Siamese network. These ML models’ performance is then evaluated on the held-out test samples of the test functional, with the resulting mean absolute errors (MAEs) denoting the difference between predicted and DFT-calculated adsorption energies.

Our primary objective in training the Siamese network on these ensemble functional energies is to learn molecular representations that capture the inherent similarities and variances between pairs of molecules across different BEEF-vdW ensemble energies. The effectiveness of these learned representations is validated by using them to train ML models that predict the adsorption energies on distinct DFT functionals.

#### Representations Generated from Flat Fingerprints

To assess the performance of our approach in the context of the BEEF-vdW ensemble model (BEM) training, we first evaluated the performance of the model using flat molecular fingerprints. Table 4 presents the corresponding outcomes for this experiment. In this table, we also present the test functional used in the experimental case (Test Functional), the machine learning algorithm utilized for predicting adsorption energies (ML Alg.), and the type of representations (Original, PCA, and IMR) that were used as input for the predictive models.

**Table 4:** Evaluation of Three Molecular Representations (Original, PCA, and IMR) Generated Using 24-Length Flat Molecular Fingerprints with the BEM Training Strategy^a^

test functional	ML Alg.	original	PCA	IMR
PBE-D3	ridge	0.31 ± 0.04	0.32 ± 0.04	**0.25 ± 0.04**
	elastic	0.33 ± 0.04	0.34 ± 0.05	**0.26 ± 0.05**
	krr	0.35 ± 0.05	0.33 ± 0.06	**0.23 ± 0.05**
	svr	0.31 ± 0.06	0.30 ± 0.06	**0.24 ± 0.04**
BEEF-vdW	ridge	0.31 ± 0.05	0.31 ± 0.05	**0.19 ± 0.03**
	elastic	0.32 ± 0.05	0.33 ± 0.04	**0.19 ± 0.03**
	krr	0.33 ± 0.04	0.35 ± 0.04	**0.13 ± 0.04**
	svr	0.31 ± 0.05	0.31 ± 0.06	**0.14 ± 0.05**
RPBE	ridge	0.31 ± 0.05	0.31 ± 0.05	**0.25 ± 0.07**
	elastic	0.33 ± 0.05	0.33 ± 0.04	**0.23 ± 0.06**
	krr	0.35 ± 0.05	0.36 ± 0.04	**0.19 ± 0.04**
	svr	0.32 ± 0.07	0.34 ± 0.07	**0.21 ± 0.07**
SCAN + rVV10	ridge	0.37 ± 0.05	0.38 ± 0.04	**0.24 ± 0.05**
	elastic	0.40 ± 0.04	0.39 ± 0.05	**0.23 ± 0.05**
	krr	0.42 ± 0.08	0.41 ± 0.07	**0.20 ± 0.04**
	svr	0.38 ± 0.08	0.39 ± 0.10	**0.22 ± 0.04**

a Presented values are mean absolute errors (MAEs) between the predicted and DFT-calculated adsorption energies in electron volts (eV). A smaller MAE value indicates better performance. Values that are statistically significant based on the *t*-test are represented in bold.

As can be seen, the molecular representations generated by the Siamese network (IMR) exhibit noteworthy improvements for all testing functionals and across all predictive machine learning algorithms, as evidenced by the results. However, when our analysis was compared to the findings presented in the section on FFM training with 24-length flat fingerprints, we found no statistically significant difference in the IMR performance between the FFM and BEM training strategies for the majority of cases; specifically, this was true in 12 out of 16 cases.

#### Representations Generated from Transfer Learning

Next, in Table 5, we present the empirical results of the case study, in which we used a similar training strategy (BEM training), but with representations generated by fingerprints from the pretrained chEMBL model. Consistent with our earlier experimental findings, our current results confirm the superiority of IMR over PCA-based representations across all experimental cases. Notably, IMR showed exceptional performance specific to the BEEF-vdW functional, compared to the other three DFT functionals. We attribute this outcome to the training strategy of our Siamese model, which is based on the random BEEF-vdW ensembles.

**Table 5:** Performance Evaluation of Three Molecular Representations (Original, PCA, and IMR) Using 768-Length Fingerprints Derived from the chEMBL Model, Combined with the BEM Training Strategy^a^

test functional	ML Alg.	original	PCA	IMR
PBE-D3	ridge	0.39 ± 0.06	0.35 ± 0.05	**0.26 ± 0.04**
	elastic	0.32 ± 0.07	0.32 ± 0.04	**0.22 ± 0.04**
	krr	0.27 ± 0.07	0.29 ± 0.04	**0.22 ± 0.05**
	svr	0.28 ± 0.06	0.29 ± 0.06	**0.20 ± 0.06**
BEEF-vdW	ridge	0.42 ± 0.07	0.37 ± 0.06	**0.13 ± 0.05**
	elastic	0.34 ± 0.05	0.33 ± 0.04	**0.13 ± 0.05**
	krr	0.34 ± 0.06	0.33 ± 0.04	**0.11 ± 0.05**
	svr	0.32 ± 0.05	0.34 ± 0.05	**0.15 ± 0.07**
RPBE	ridge	0.46 ± 0.08	0.38 ± 0.03	**0.17 ± 0.04**
	elastic	0.37 ± 0.05	0.38 ± 0.04	**0.17 ± 0.03**
	krr	0.44 ± 0.07	0.37 ± 0.04	**0.18 ± 0.04**
	svr	0.38 ± 0.05	0.39 ± 0.04	**0.16 ± 0.06**
SCAN + rVV10	ridge	0.44 ± 0.06	0.38 ± 0.04	**0.25 ± 0.10**
	elastic	0.39 ± 0.05	0.39 ± 0.03	**0.23 ± 0.07**
	krr	0.37 ± 0.04	0.39 ± 0.03	**0.18 ± 0.05**
	svr	0.35 ± 0.05	0.39 ± 0.05	**0.20 ± 0.04**

a The values are given in terms of mean absolute errors (MAEs) between the predicted and DFT-calculated adsorption energies, expressed in electron volts (eV). A lower MAE value signifies superior performance. Bolded values indicate statistical significance, as determined by *t*-test.

#### Representations Generated from Morgan Fingerprints

Diving into the results from the Morgan fingerprints, as presented in Table 6, our application of the BEM training strategy again demonstrated patterns that were in line with our prior observations. Much like before, the IMR consistently outperformed the PCA-based representations across all of the experimental cases. Specifically, when using the BEEF-vdW functional for testing, the performance spike was evident. As mentioned in the earlier section, this enhanced performance, particularly to the BEEF-vdW test functional, can likely be traced back to our Siamese model’s training approach, which heavily leverages the random BEEF-vdW ensembles.

**Table 6:** Performance Evaluation of Three Molecular Representations (Original, PCA, and IMR) Using 24-Length Morgan Fingerprints with the BEM Training Strategy^a^

test functional	ML Alg.	original	PCA	IMR
PBE-D3	ridge	0.34 ± 0.05	0.33 ± 0.04	0.31 ± 0.07
	elastic	0.32 ± 0.05	0.31 ± 0.04	**0.22 ± 0.04**
	krr	0.31 ± 0.05	0.34 ± 0.06	**0.27 ± 0.06**
	svr	0.31 ± 0.05	0.32 ± 0.06	**0.25 ± 0.05**
BEEF-vdW	ridge	0.34 ± 0.04	0.34 ± 0.05	**0.11 ± 0.02**
	elastic	0.33 ± 0.03	0.33 ± 0.04	**0.12 ± 0.03**
	krr	0.33 ± 0.04	0.34 ± 0.04	**0.10 ± 0.05**
	svr	0.33 ± 0.05	0.33 ± 0.05	**0.10 ± 0.07**
RPBE	ridge	0.37 ± 0.06	0.37 ± 0.07	**0.18 ± 0.09**
	elastic	0.37 ± 0.05	0.39 ± 0.05	**0.19 ± 0.13**
	krr	0.38 ± 0.05	0.38 ± 0.05	**0.18 ± 0.06**
	svr	0.38 ± 0.06	0.38 ± 0.06	**0.18 ± 0.05**
SCAN + rVV10	ridge	0.39 ± 0.05	0.39 ± 0.05	**0.23 ± 0.06**
	elastic	0.40 ± 0.03	0.39 ± 0.04	**0.22 ± 0.05**
	krr	0.39 ± 0.04	0.39 ± 0.03	**0.19 ± 0.03**
	svr	0.39 ± 0.05	0.39 ± 0.05	**0.22 ± 0.05**

a The values are presented in terms of mean absolute errors (MAEs) between the predicted and DFT-calculated adsorption energies, expressed in electron volts (eV). A lower MAE value denotes a superior performance. Values highlighted in bold are determined to be statistically significant via *t*-test.

### Sanity Check

As a validation of our approach, we have conducted four experimental case studies using the four DFT functionals (similar to FFM and BEM) but these case studies are designed to simulate the scenario of model training without additional functionals; the results are presented in detail in the Supporting Information, which includes information on the experimental setup and the corresponding findings.

### Fingerprint Contribution Analysis

**Figure 4:**
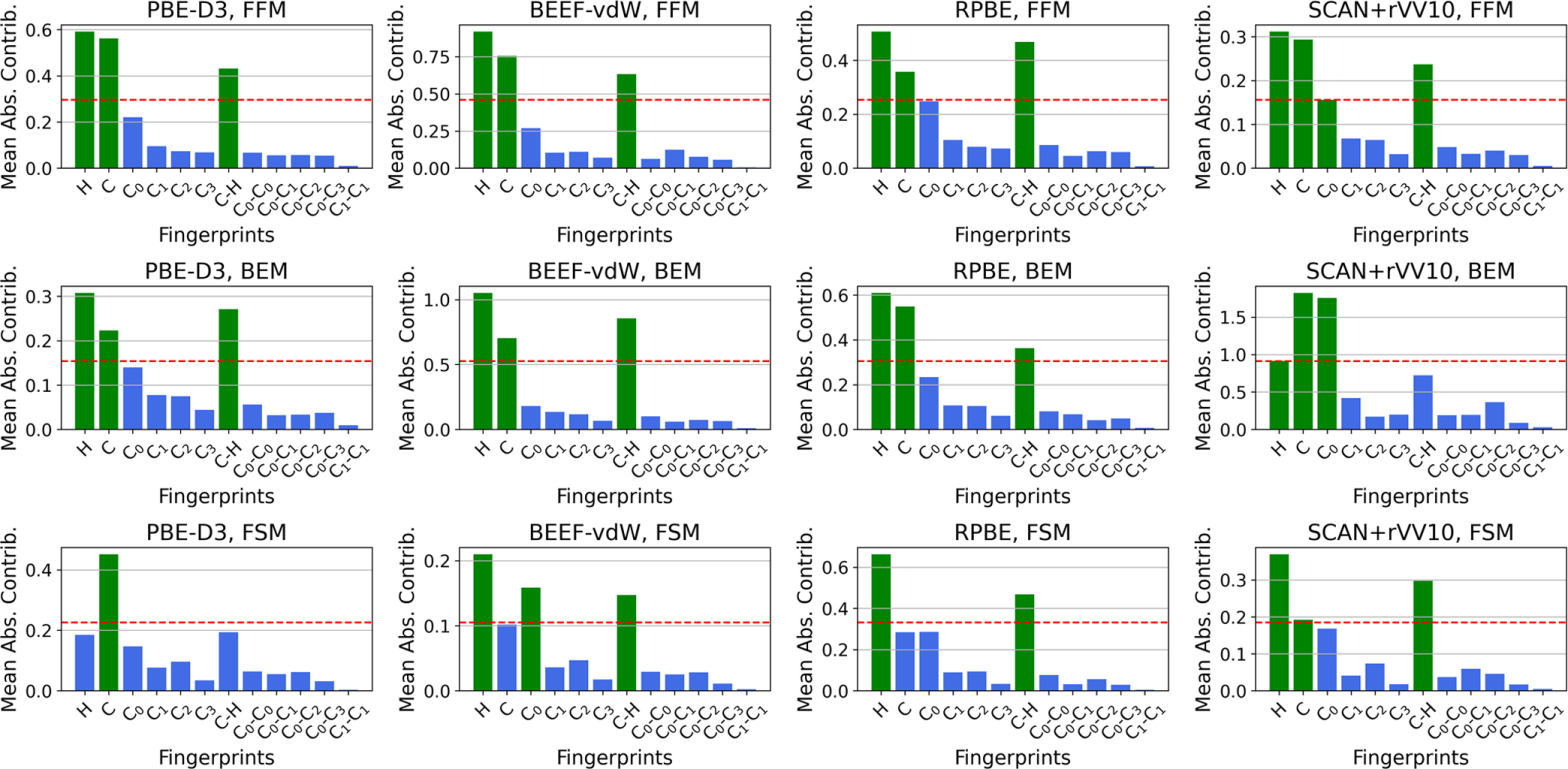
Feature contribution analysis across different training strategies and DFT functionals. This figure illustrates the mean absolute contribution of various molecular fingerprints (e.g., H, C, and C_0_) in a matrix format, where rows and columns represent training strategies for the Siamese network and DFT functionals, respectively. The first, second, and third rows represent the FFM, BEM, and FSM training strategies, while the first to fourth columns correspond to PBE-D3, BEEF-vdW, RPBE, and SCAN + rVV10 functionals, respectively. A dotted red line in each plot marks a threshold set at 50% of the maximum contribution value for that specific scenario, delineating the top contributing fingerprints. Fingerprints with negligible contributions were omitted for clarity. This analysis underscores the significant fingerprints contributing to adsorption energies across various training strategies and functionals.

In this section, we present a comprehensive feature contribution analysis to examine the impact of different molecular fingerprints on the adsorption energy predictions. Our analysis includes all three training strategies for the Siamese network, namely, the four functional model (FFM), the BEEF-vdW ensemble model (BEM), and the functional specific model (FSM), across all four distinct DFT functionals: PBE-D3, BEEF-vdW, RPBE, and SCAN + rVV10. Figure 4 illustrates the mean absolute contribution of the fingerprints (such as H, C, and C_0_) for each training strategy and functional. The attribution values shown here were determined using integrated gradients, a method implemented in the Python-based Captum library.^58^ Notably, for functionals like PBE-D3, BEEF-vdW, and RPBE, both FFM and BEM consistently identify the same top contributing fingerprints (e.g., number of hydrogen atoms, number of carbon atoms, and number of carbon–hydrogen bonds), indicating a shared understanding of key features. This agreement highlights the robustness of the proposed training strategy to learn the IMR that captures essential characteristics from the original fingerprints, leading to superior predictive performance. However, the FSM strategy, which lacks the benefit of leveraging additional functionals, shows divergent feature contributions, underlining the effectiveness of FFM and BEM in exploiting functional invariances. Interestingly, when testing on the SCAN + rVV10 functional, we see weaker agreement between FFM and BEM. This can be attributed to the distinct characteristics of SCAN + rVV10 as a meta-GGA functional, in contrast to the other three GGA (generalized gradient approximation) functionals (such as PBE-D3, BEEF-vdW, and RPBE) used in obtaining the invariant molecular representations, highlighting the importance of using diverse functionals in generating the invariant and robust representations. Notably, this aligns with findings by a prior study,^59^ which demonstrated through Mahalanobis distance analysis that SCAN + rVV10 is comparatively less accurately captured by methods typically effective for GGA functionals, underscoring its unique functional properties. The fingerprint contribution analysis emphasizes the capability of our proposed strategies to extract meaningful essence and insights from original molecular fingerprints, thereby generating superior representations that ultimately enhance the predictive accuracy of the adsorption energies. A species-based breakdown of fingerprint contributions is provided in the Supporting Information for one of the experimental case scenarios.

### Goodness-of-Fit Analysis (Using *D*^2^-Score)

Finally, we conducted a comprehensive goodness-of-fit analysis using the *D*^2^-score, implemented in Python’s Scikit-learn^60^ library. The *D*^2^-score is a metric that assesses how well a model explains variance compared to a null model, where the null model is based on using the median from the training samples as the predictions for the test samples. Additional details on how this score is defined can be found in the Supporting Information. This approach was utilized to evaluate the performance of our models across all three training strategies, namely, FFM, BEM, and FSM, along with the molecular representations used in the study, Original, PCA, and IMR. Our findings reveal that models based on Original and PCA representations were no better than the null model, a behavior observed irrespective of molecular fingerprints used, such as 24-length flat molecular fingerprints, 768-length chEMBL fingerprints, and 24-length Morgan fingerprints, for generating molecular representations. This phenomenon highlights the challenges posed by the data set size and complexity of the molecular interactions considered in our study. In contrast, models trained with the IMR consistently led to significantly better-fitted models, in the case of FFM and BEM training strategies, underscoring the benefit of leveraging additional functionals to learn robust representations. Furthermore, aligning with our prior findings, we observed that models especially demonstrate a better fit to the BEEF-vdW functional when tested on both the FFM and BEM training strategies. Detailed results, including the *D*^2^-scores for each case study, are available in the Supporting Information for further reference.

## Conclusions

In our proposed study, we introduced a novel learning method for predicting the adsorption energies of reaction intermediates. Our approach demonstrates the efficacy of learning from multiple functionals to overcome the challenges posed by the idiosyncrasies of different functionals by capturing the relative energy difference between pairs of intermediates calculated within the same functional and training the model across all different functionals. The present study has reached several key conclusions: (i) by incorporating additional functionals, our proposed method generates superior representations (IMR) that lead to significantly improved performance compared to the Original and PCA-based representations; (ii) throughout numerous test cases, our Siamese model consistently performed more effectively with BEEF-vdW as the test functional. Interestingly, this boost in performance persisted not only in the BEM training strategy, where we used BEEF-vdW ensembles to train the Siamese model, but also when the model was trained on the other three DFT functionals (FFM training strategy); and finally, (iii) the performance of two different training strategies with additional functionals (FFM and BEM) was found to be similar, despite the fact that FFM employed only three training DFT functionals, whereas the BEM employed 50 random ensembles or functionals. This can be attributed to the fact that even though the number of BEEF-vdW ensembles employed in the training process is large, they are less diverse compared to the DFT functionals, resulting in each DFT functional being more informative for learning the invariant molecular representations (IMR) of the species.

Our proposed approach represents a significant advancement in the field of predicting the adsorption energy of reaction intermediates, as it enables the capture of the underlying chemistry of the system in a manner that is insensitive to the choice of functional and aware of the system on which the models are trained on. The findings of our study highlight the potential of our approach to generating informative and robust molecular representations, which can result in improved performance in predictive modeling tasks.

Moving forward, we anticipate the general applicability of our method to a broad spectrum of functionals and catalyst surfaces. Our future work aims to further validate and reinforce our approach by applying it to a larger data set of functionals, catalyst surfaces, and reaction intermediates. In addition, a key area of potential work is predicting reliable transition state energies through machine learning, as these are often the most time-consuming steps for generating accurate chemical reaction models on catalysts. Incorporating these transition states and reaction energies to generate better predictive models can help to generate accurate inputs with quantified uncertainty into microkinetic models, to better predict key experimental data, and to allow for accurate calibration of existing experimental data into kinetic models. The objective is to learn molecular representations that are invariant, robust, and reliable, which can inform the design and optimization of catalysts for chemical reactions. We expect that our proposed method holds great promise in the field of predictive modeling for the adsorption energy of reaction intermediates and has the potential to make a substantial impact in this area.

## Data Availability

The data used in this research, including 46 intermediate species, their SMILES notations, and adsorption energies for the Pt(111) metal surface calculated using four DFT functionals and the 2000 BEEF-vdW ensembles, are provided as part of the Supporting Information. This data was collected and prepared using the Vienna Ab initio Simulation Package (VASP) version 5.4.4, details of which can be found at https://vasp.at. The pretrained chEMBL model used to generate the 768-length chEMBL molecular fingerprints can be accessed at https://huggingface.co/mrm8488/chEMBL_smiles_v1.

## Supporting Information Available

Contains additional results, namely, sanity check with functional specific model (FSM); (a) prediction results for three types of molecular representations (Original, PCA, and IMR) generated by 24-length flat molecular fingerprints; (b) prediction results for three types of molecular representations (Original, PCA, and IMR) generated by a pretrained chEMBL model; (c) prediction results for three types of molecular representations (Original, PCA, and IMR) generated by 24-length Morgan fingerprints; detailed information on DFT calculations; species-based fingerprint contribution analysis for a specific case study; goodness-of-fit analysis for each case study using the *D*^2^-score; tabular format data set containing molecular species, their SMILES notations, and adsorption energies for DFT functionals (PDF)
IMR—data files (ZIP)

## References

[ref1] Catlow C. R.; Davidson M.; Hardacre C.; Hutchings G. J. Catalysis making the world a better place. Philos. Trans. R. Soc., A 2016, 374, 20150089 10.1098/rsta.2015.0089. PMC4707691 26755766

[ref2] Reuter K.; Plaisance C. P.; Oberhofer H.; Andersen M. Perspective: On the active site model in computational catalyst screening. J. Chem. Phys. 2017, 146, 040901 10.1063/1.4974931. 28147553

[ref3] Jover J.; Fey N. The computational road to better catalysts. Chem. - Asian J. 2014, 9, 1714–1723. 10.1002/asia.201301696. 24668590

[ref4] Nørskov J. K.; Studt F.; Abild-Pedersen F.; Bligaard T. Fundamental Concepts in Heterogeneous Catalysis; John Wiley & Sons, 2014.

[ref5] Motagamwala A. H.; Dumesic J. A. Microkinetic modeling: a tool for rational catalyst design. Chem. Rev. 2021, 121, 1049–1076. 10.1021/acs.chemrev.0c00394. 33205961

[ref6] Bo C.; Maseras F.; López N. The role of computational results databases in accelerating the discovery of catalysts. Nat. Catal. 2018, 1, 809–810. 10.1038/s41929-018-0176-4.

[ref7] Nørskov J. K.; Abild-Pedersen F.; Studt F.; Bligaard T. Density functional theory in surface chemistry and catalysis. Proc. Natl. Acad. Sci. U.S.A. 2011, 108, 937–943. 10.1073/pnas.1006652108. 21220337 PMC3024687

[ref8] Busch M.; Wodrich M. D.; Corminboeuf C. Linear scaling relationships and volcano plots in homogeneous catalysis-revisiting the Suzuki reaction. Chem. Sci. 2015, 6, 6754–6761. 10.1039/C5SC02910D. 28757966 PMC5508671

[ref9] Abild-Pedersen F.; Greeley J.; Studt F.; Rossmeisl J.; Munter T. R.; Moses P. G.; Skulason E.; Bligaard T.; Nørskov J. K. Scaling properties of adsorption energies for hydrogen-containing molecules on transition-metal surfaces. Phys. Rev. Lett. 2007, 99, 016105 10.1103/PhysRevLett.99.016105. 17678168

[ref10] Greeley J. Theoretical heterogeneous catalysis: scaling relationships and computational catalyst design. Annu. Rev. Chem. Biomol. Eng. 2016, 7, 605–635. 10.1146/annurev-chembioeng-080615-034413. 27088666

[ref11] Chowdhury A. J.; Yang W.; Walker E.; Mamun O.; Heyden A.; Terejanu G. A. Prediction of adsorption energies for chemical species on metal catalyst surfaces using machine learning. J. Phys. Chem. C 2018, 122, 28142–28150. 10.1021/acs.jpcc.8b09284.

[ref12] Bishop C. M. Pattern Recognition and Machine Learning; Springer, 2006.

[ref13] Lopez-Paz D.; Sra S.; Smola A.; Ghahramani Z.; Schölkopf B. In Randomized Nonlinear Component Analysis, International Conference on Machine Learning; MLResearchPress, 2014; pp 1359–1367.

[ref14] Behler J.; Parrinello M. Generalized neural-network representation of high-dimensional potential-energy surfaces. Phys. Rev. Lett. 2007, 98, 146401 10.1103/PhysRevLett.98.146401. 17501293

[ref15] Pereira F.; Xiao K.; Latino D. A.; Wu C.; Zhang Q.; Aires-de Sousa J. Machine learning methods to predict density functional theory B3LYP energies of HOMO and LUMO orbitals. J. Chem. Inf. Model. 2017, 57, 11–21. 10.1021/acs.jcim.6b00340. 28033004

[ref16] Chowdhury A. J.; Yang W.; Abdelfatah K. E.; Zare M.; Heyden A.; Terejanu G. A. A multiple filter based neural network approach to the extrapolation of adsorption energies on metal surfaces for catalysis applications. J. Chem. Theory Comput. 2020, 16, 1105–1114. 10.1021/acs.jctc.9b00986. 31962041

[ref17] Chowdhury A. J.; Yang W.; Heyden A.; Terejanu G. A. Comparative Study on the Machine Learning-Based Prediction of Adsorption Energies for Ring and Chain Species on Metal Catalyst Surfaces. J. Phys. Chem. C 2021, 125, 17742–17748. 10.1021/acs.jpcc.1c05470.

[ref18] Kolluru A.; Shuaibi M.; Palizhati A.; Shoghi N.; Das A.; Wood B.; Zitnick C. L.; Kitchin J. R.; Ulissi Z. W. Open Challenges in Developing Generalizable Large-Scale Machine-Learning Models for Catalyst Discovery. ACS Catal. 2022, 12, 8572–8581. 10.1021/acscatal.2c02291.

[ref19] Chanussot L.; Das A.; Goyal S.; Lavril T.; Shuaibi M.; Riviere M.; Tran K.; Heras-Domingo J.; Ho C.; Hu W.; et al. Open catalyst 2020 (OC20) dataset and community challenges. ACS Catal. 2021, 11, 6059–6072. 10.1021/acscatal.0c04525.

[ref20] Ben-David S.; Blitzer J.; Crammer K.; Kulesza A.; Pereira F.; Vaughan J. W. A theory of learning from different domains. Mach. Learn. 2010, 79, 151–175. 10.1007/s10994-009-5152-4.

[ref21] Medford A. J.; Wellendorff J.; Vojvodic A.; Studt F.; Abild-Pedersen F.; Jacobsen K. W.; Bligaard T.; Nørskov J. K. Assessing the reliability of calculated catalytic ammonia synthesis rates. Science 2014, 345, 197–200. 10.1126/science.1253486. 25013071

[ref22] Wellendorff J.; Lundgaard K. T.; Møgelhøj A.; Petzold V.; Landis D. D.; Nørskov J. K.; Bligaard T.; Jacobsen K. W. Density functionals for surface science: Exchange-correlation model development with Bayesian error estimation. Phys. Rev. B 2012, 85, 235149 10.1103/PhysRevB.85.235149.

[ref23] Rupp M.; Tkatchenko A.; Müller K.-R.; Von Lilienfeld O. A. Fast and accurate modeling of molecular atomization energies with machine learning. Phys. Rev. Lett. 2012, 108, 058301 10.1103/PhysRevLett.108.058301. 22400967

[ref24] Hansen K.; Biegler F.; Ramakrishnan R.; Pronobis W.; Von Lilienfeld O. A.; Muller K.-R.; Tkatchenko A. Machine learning predictions of molecular properties: Accurate many-body potentials and nonlocality in chemical space. J. Phys. Chem. Lett. 2015, 6, 2326–2331. 10.1021/acs.jpclett.5b00831. 26113956 PMC4476293

[ref25] Behler J. Atom-centered symmetry functions for constructing high-dimensional neural network potentials. J. Chem. Phys. 2011, 134, 074106 10.1063/1.3553717. 21341827

[ref26] Morawietz T.; Behler J. A density-functional theory-based neural network potential for water clusters including van der Waals corrections. J. Phys. Chem. A 2013, 117, 7356–7366. 10.1021/jp401225b. 23557541

[ref27] Behler J. Perspective: Machine learning potentials for atomistic simulations. J. Chem. Phys. 2016, 145, 170901 10.1063/1.4966192. 27825224

[ref28] Ulissi Z. W.; Tang M. T.; Xiao J.; Liu X.; Torelli D. A.; Karamad M.; Cummins K.; Hahn C.; Lewis N. S.; Jaramillo T. F.; et al. Machine-learning methods enable exhaustive searches for active bimetallic facets and reveal active site motifs for CO2 reduction. ACS Catal. 2017, 7, 6600–6608. 10.1021/acscatal.7b01648.

[ref29] Rogers D.; Hahn M. Extended-connectivity fingerprints. J. Chem. Inf. Model. 2010, 50, 742–754. 10.1021/ci100050t. 20426451

[ref30] Lo Y.-C.; Rensi S. E.; Torng W.; Altman R. B. Machine learning in chemoinformatics and drug discovery. Drug Discovery Today 2018, 23, 1538–1546. 10.1016/j.drudis.2018.05.010. 29750902 PMC6078794

[ref31] Sanchez-Lengeling B.; Aspuru-Guzik A. Inverse molecular design using machine learning: Generative models for matter engineering. Science 2018, 361, 360–365. 10.1126/science.aat2663. 30049875

[ref32] Wu Z.; Ramsundar B.; Feinberg E. N.; Gomes J.; Geniesse C.; Pappu A. S.; Leswing K.; Pande V. MoleculeNet: a benchmark for molecular machine learning. Chem. Sci. 2018, 9, 513–530. 10.1039/C7SC02664A. 29629118 PMC5868307

[ref33] Kearnes S.; McCloskey K.; Berndl M.; Pande V.; Riley P. Molecular graph convolutions: moving beyond fingerprints. J. Comput.-Aided Mol. Des. 2016, 30, 595–608. 10.1007/s10822-016-9938-8. 27558503 PMC5028207

[ref34] Duvenaud D. K.; Maclaurin D.; Iparraguirre J.; Bombarell R.; Hirzel T.; Aspuru-Guzik A.; Adams R. P. Convolutional Networks on Graphs for Learning Molecular Fingerprints, Advances in Neural Information Processing Systems; NIPS, 2015.

[ref35] Rupp M.; Ramakrishnan R.; Von Lilienfeld O. A. Machine learning for quantum mechanical properties of atoms in molecules. J. Phys. Chem. Lett. 2015, 6, 3309–3313. 10.1021/acs.jpclett.5b01456.

[ref36] Segler M. H. S.; Kogej T.; Tyrchan C.; Waller M. P. Generating focused molecule libraries for drug discovery with recurrent neural networks. ACS Cent. Sci. 2018, 4, 120–131. 10.1021/acscentsci.7b00512. 29392184 PMC5785775

[ref37] Torng W.; Altman R. B. 3D deep convolutional neural networks for amino acid environment similarity analysis. BMC Bioinf. 2017, 18, 302 10.1186/s12859-017-1702-0. PMC5472009 28615003

[ref38] Chopra S.; Hadsell R.; LeCun Y. Learning a Similarity Metric Discriminatively, with Application to Face Verification, 2005 IEEE Computer Society Conference on Computer Vision and Pattern Recognition (CVPR’05); IEEE, 2005; pp 539–546.

[ref39] Bromley J.; Guyon I.; LeCun Y.; Säckinger E.; Shah R. Signature Verification Using a ”Siamese” Time Delay Neural Network, Advances in Neural Information Processing System; NIPS, 1993.

[ref40] Fricke C.; Rajbanshi B.; Walker E. A.; Terejanu G.; Heyden A. Propane Dehydrogenation on Platinum Catalysts: Identifying the Active Sites through Bayesian Analysis. ACS Catal. 2022, 12, 2487–2498. 10.1021/acscatal.1c04844.

[ref41] Perdew J. P.; Burke K.; Ernzerhof M. Generalized gradient approximation made simple. Phys. Rev. Lett. 1996, 77, 3865 10.1103/PhysRevLett.77.3865. 10062328

[ref42] Grimme S.; Antony J.; Ehrlich S.; Krieg H. A consistent and accurate ab initio parametrization of density functional dispersion correction (DFT-D) for the 94 elements H-Pu. J. Chem. Phys. 2010, 132, 154104 10.1063/1.3382344. 20423165

[ref43] Hammer B.; Hansen L. B.; Nørskov J. K. Improved adsorption energetics within density-functional theory using revised Perdew-Burke-Ernzerhof functionals. Phys. Rev. B 1999, 59, 7413 10.1103/PhysRevB.59.7413.

[ref44] Peng H.; Yang Z.-H.; Perdew J. P.; Sun J. Versatile van der Waals density functional based on a meta-generalized gradient approximation. Phys. Rev. X 2016, 6, 041005 10.1103/PhysRevX.6.041005.

[ref45] Kresse G.; Furthmüller J. Efficiency of ab-initio total energy calculations for metals and semiconductors using a plane-wave basis set. Comput. Mater. Sci. 1996, 6, 15–50. 10.1016/0927-0256(96)00008-0. 9984901

[ref46] Kresse G.; Furthmüller J. Efficient iterative schemes for ab initio total-energy calculations using a plane-wave basis set. Phys. Rev. B 1996, 54, 11169 10.1103/PhysRevB.54.11169. 9984901

[ref47] Kresse G.; Hafner J. Ab initio molecular dynamics for liquid metals. Phys. Rev. B 1993, 47, 558 10.1103/PhysRevB.47.558. 10004490

[ref48] Collins C. R.; Gordon G. J.; Von Lilienfeld O. A.; Yaron D. J. Constant size descriptors for accurate machine learning models of molecular properties. J. Chem. Phys. 2018, 148, 241718 10.1063/1.5020441. 29960361

[ref49] chEMBL pretrained model. https://huggingface.co/mrm8488/chEMBL_smiles_v1.

[ref50] Gupta A.; Müller A. T.; Huisman B. J.; Fuchs J. A.; Schneider P.; Schneider G. Generative recurrent networks for de novo drug design. Mol. Inform. 2018, 37, 1700111 10.1002/minf.201700111. 29095571 PMC5836943

[ref51] Morgan H. L. The generation of a unique machine description for chemical structures-a technique developed at chemical abstracts service. J. Chem. Doc. 1965, 5, 107–113. 10.1021/c160017a018.

[ref52] Karlik B.; Olgac A. V. Performance analysis of various activation functions in generalized MLP architectures of neural networks.. Int. J. Artif. Intell. Expert Syst. 2011, 1, 111–122.

[ref53] Tan T. G.; Teo J.; Anthony P. A comparative investigation of non-linear activation functions in neural controllers for search-based game AI engineering. Artif. Intell. Rev. 2014, 41, 1–25. 10.1007/s10462-011-9294-y.

[ref54] Maas A. L.; Hannun A. Y.; Ng A. Y. Rectifier Nonlinearities Improve Neural Network Acoustic Models, Proc. ICML; ICML, 2013.

[ref55] LeCun Y.; Bottou L.; Bengio Y.; Haffner P. Gradient-based learning applied to document recognition. Proc. IEEE 1998, 86, 2278–2324. 10.1109/5.726791.

[ref56] Sutskever I.; Martens J.; Dahl G.; Hinton G. On the importance of initialization and momentum in deep learning. Mach. Learn. 2013, 1139–1147.

[ref57] Hanin B.; Rolnick D. How to Start Training: The Effect of Initialization and Architecture, Advances in Neural Information Processing Systems; NIPS, 2018.

[ref58] Kokhlikyan N.; Miglani V.; Martin M.; Wang E.; Alsallakh B.; Reynolds J.; Melnikov A.; Kliushkina N.; Araya C.; Yan S.; Reblitz-Richardson O. Captum: A unified and generic model interpretability library for Pytorch. 2020, arXiv:2009.07896. arXiv.org e-Print archive. https://arxiv.org/abs/2009.07896.

[ref59] Szaro N. A.; Bello M.; Fricke C. H.; Bamidele O. H.; Heyden A. Benchmarking the accuracy of density functional theory against the random phase approximation for the ethane dehydrogenation network on Pt (111). J. Phys. Chem. Lett. 2023, 14, 10769–10778. 10.1021/acs.jpclett.3c02723. 38011289

[ref60] Pedregosa F.; Varoquaux G.; Gramfort A.; Michel V.; Thirion B.; Grisel O.; Blondel M.; Prettenhofer P.; Weiss R.; Dubourg V.; Vanderplas J. Scikit-learn: Machine Learning in Python. J. Mach. Learn. Res. 2011, 12, 2825–2830.

