# Supplementary material for: Invariant Molecular Representations for Heterogeneous Catalysis
Source: J Chem Inf Model. 2024 Jan 10;64(2):327–39. doi: 10.1021/acs.jcim.3c00594 (PMC10806804; doi:10.1021/acs.jcim.3c00594)
Supplement: Supplementary file 1 — ci3c00594_si_001.pdf [file ci3c00594_si_001.pdf]

Supporting Information

Invariant Molecular Representations for Heterogeneous Catalysis

Jawad Chowdhury,† Charles Fricke,‡ Olajide Bamidele,‡ Mubarak Bello,‡ Wenqiang Yang,‡ Andreas Heyden,*,‡ and Gabriel Terejanu*,†

†*Department of Computer Science, University of North Carolina at Charlotte, Charlotte, North Carolina 28223, USA*

‡*Department of Chemical Engineering, University of South Carolina, Columbia, South Carolina 29208, USA*

E-mail: heyden@cec.sc.edu; gterejan@uncc.edu

Sanity Check - Functional Specific Model (FSM)

In this section, we conducted four experimental case studies for the four DFT functionals (similar to the FFM and BEM). However, in all these cases, we have used both the training samples for the Siamese network and samples for the predictive analysis from the same functional. This simulates the scenario in which we do not have access to information from additional functionals but the Siamese model training is specific to the particular functional where it is tested.

Representations Generated from Flat Fingerprints

Table S1: Performance evaluation of three molecular representation types (Original, PCA, and IMR) derived from 24-length flat molecular fingerprints using the FSM training approach. Values are given as Mean Absolute Errors (MAEs) between the predicted and DFT-calculated adsorption energies, expressed in electron volts (eV). A lower MAE signifies enhanced performance.

Test Functional	ML Alg.	Original	PCA	IMR
PBE-D3	ridge	0.31 ± 0.04	0.32 ± 0.04	0.33 ± 0.04
	elastic	0.33 ± 0.04	0.34 ± 0.05	0.33 ± 0.04
	krr	0.35 ± 0.05	0.33 ± 0.05	0.38 ± 0.10
	svr	0.31 ± 0.06	0.30 ± 0.06	0.36 ± 0.08
BEEF-vdW	ridge	0.31 ± 0.05	0.31 ± 0.05	0.32 ± 0.04
	elastic	0.32 ± 0.05	0.33 ± 0.04	0.32 ± 0.04
	krr	0.33 ± 0.04	0.35 ± 0.04	0.37 ± 0.10
	svr	0.31 ± 0.05	0.31 ± 0.06	0.37 ± 0.09
RPBE	ridge	0.31 ± 0.05	0.31 ± 0.05	0.32 ± 0.05
	elastic	0.33 ± 0.05	0.33 ± 0.04	0.33 ± 0.05
	krr	0.35 ± 0.05	0.36 ± 0.04	0.37 ± 0.11
	svr	0.32 ± 0.07	0.34 ± 0.07	0.34 ± 0.10
SCAN+rVV10	ridge	0.37 ± 0.05	0.38 ± 0.04	0.38 ± 0.04
	elastic	0.40 ± 0.04	0.39 ± 0.05	0.39 ± 0.04
	krr	0.42 ± 0.08	0.42 ± 0.07	0.41 ± 0.13
	svr	0.38 ± 0.08	0.39 ± 0.10	0.42 ± 0.12

We first present the results of our experiments using FSM training and representations generated from flat molecular fingerprints. The results are illustrated in Table S1. Our empirical findings show no significant difference in the performance of molecular representations

using our proposed model (IMR) compared to the PCA-based representations (PCA). This suggests that even in the absence of information from additional functionals, the performance of IMR is on par with that of baseline representations.

Representations Generated from Transfer Learning

Table S2: Performance assessment of three molecular representation types (Original, PCA, and IMR) derived from fingerprints of the pretrained chEMBL model via FSM training. Values are presented as Mean Absolute Errors (MAEs) between predicted and DFT-calculated adsorption energies, in electron volts (eV). A lower MAE suggests superior accuracy.

Test Functional	ML Alg.	Original	PCA	IMR
PBE-D3	ridge	0.39 ± 0.06	0.35 ± 0.05	0.31 ± 0.05
	elastic	0.32 ± 0.07	0.33 ± 0.06	0.31 ± 0.05
	krr	0.27 ± 0.07	0.28 ± 0.04	0.30 ± 0.05
	svr	0.28 ± 0.06	0.29 ± 0.06	0.31 ± 0.05
BEEF-vdW	ridge	0.42 ± 0.07	0.37 ± 0.04	0.34 ± 0.04
	elastic	0.34 ± 0.05	0.33 ± 0.03	0.34 ± 0.04
	krr	0.34 ± 0.06	0.32 ± 0.04	0.34 ± 0.04
	svr	0.32 ± 0.05	0.34 ± 0.04	0.34 ± 0.05
RPBE	ridge	0.46 ± 0.08	0.39 ± 0.05	0.38 ± 0.04
	elastic	0.37 ± 0.05	0.37 ± 0.04	0.37 ± 0.03
	krr	0.44 ± 0.07	0.37 ± 0.04	0.38 ± 0.05
	svr	0.38 ± 0.05	0.40 ± 0.04	0.38 ± 0.05
SCAN+rVV10	ridge	0.44 ± 0.06	0.38 ± 0.04	0.36 ± 0.06
	elastic	0.39 ± 0.05	0.39 ± 0.03	0.36 ± 0.06
	krr	0.37 ± 0.04	0.39 ± 0.03	0.35 ± 0.06
	svr	0.35 ± 0.05	0.39 ± 0.05	0.36 ± 0.06

In Table S2, we present the results of the experimental cases with representations generated by using FSM training and chEMBL fingerprints. Again, we see no statistical difference between the IMR and the PCA representations for any of the cases. Analogous to the scenario with flat molecular fingerprints, we conclude that our proposed method generates molecular representations (IMR) that perform equally well for predictive modeling compared to the baseline methods (Original, PCA), even when the training data is specific to only one functional.

Representations Generated from Morgan Fingerprints

Table S3: Evaluation of three molecular representation types (Original, PCA, and IMR) derived from 24-length Morgan fingerprints through FSM training. The values are given as Mean Absolute Errors (MAEs) between the predicted and DFT-calculated adsorption energies, expressed in electron volts (eV). A lower MAE suggests enhanced accuracy.

Test Functional	ML Alg.	Original	PCA	IMR
PBE-D3	ridge	0.34 ± 0.05	0.33 ± 0.04	0.36 ± 0.05
	elastic	0.32 ± 0.05	0.31 ± 0.04	0.34 ± 0.05
	krr	0.31 ± 0.05	0.32 ± 0.05	0.36 ± 0.07
	svr	0.31 ± 0.05	0.31 ± 0.05	0.36 ± 0.07
BEEF-vdW	ridge	0.34 ± 0.04	0.34 ± 0.05	0.34 ± 0.04
	elastic	0.33 ± 0.03	0.33 ± 0.04	0.34 ± 0.05
	krr	0.33 ± 0.04	0.34 ± 0.04	0.34 ± 0.05
	svr	0.33 ± 0.05	0.33 ± 0.05	0.33 ± 0.05
RPBE	ridge	0.37 ± 0.06	0.38 ± 0.06	0.39 ± 0.07
	elastic	0.37 ± 0.05	0.39 ± 0.05	0.39 ± 0.07
	krr	0.38 ± 0.05	0.39 ± 0.05	0.42 ± 0.06
	svr	0.38 ± 0.06	0.38 ± 0.06	0.39 ± 0.08
SCAN+rVV10	ridge	0.39 ± 0.05	0.39 ± 0.05	0.40 ± 0.05
	elastic	0.40 ± 0.03	0.39 ± 0.04	0.41 ± 0.04
	krr	0.39 ± 0.04	0.39 ± 0.03	0.41 ± 0.05
	svr	0.39 ± 0.05	0.40 ± 0.05	0.40 ± 0.05

Turning our attention to Morgan fingerprints, the findings from our FSM training are documented in Table S3. Again we see the trend persists. The comparative evaluation between our proposed IMR model and PCA representations reveals no significant differences. Thus, it's evident that, even when utilizing Morgan fingerprints, the IMR continues to deliver performance on par with established baselines in the absence of additional functionals.

DFT Calculation Details

All DFT calculations were performed using the Vienna Ab initio Simulation Package (VASP) version 5.4.4. For geometric optimization, electron-exchange correlation was described by the Perdew-Burke-Ernzerhof (PBE)[1] functional coupled with dispersion corrections based on the D3-technique.[2]

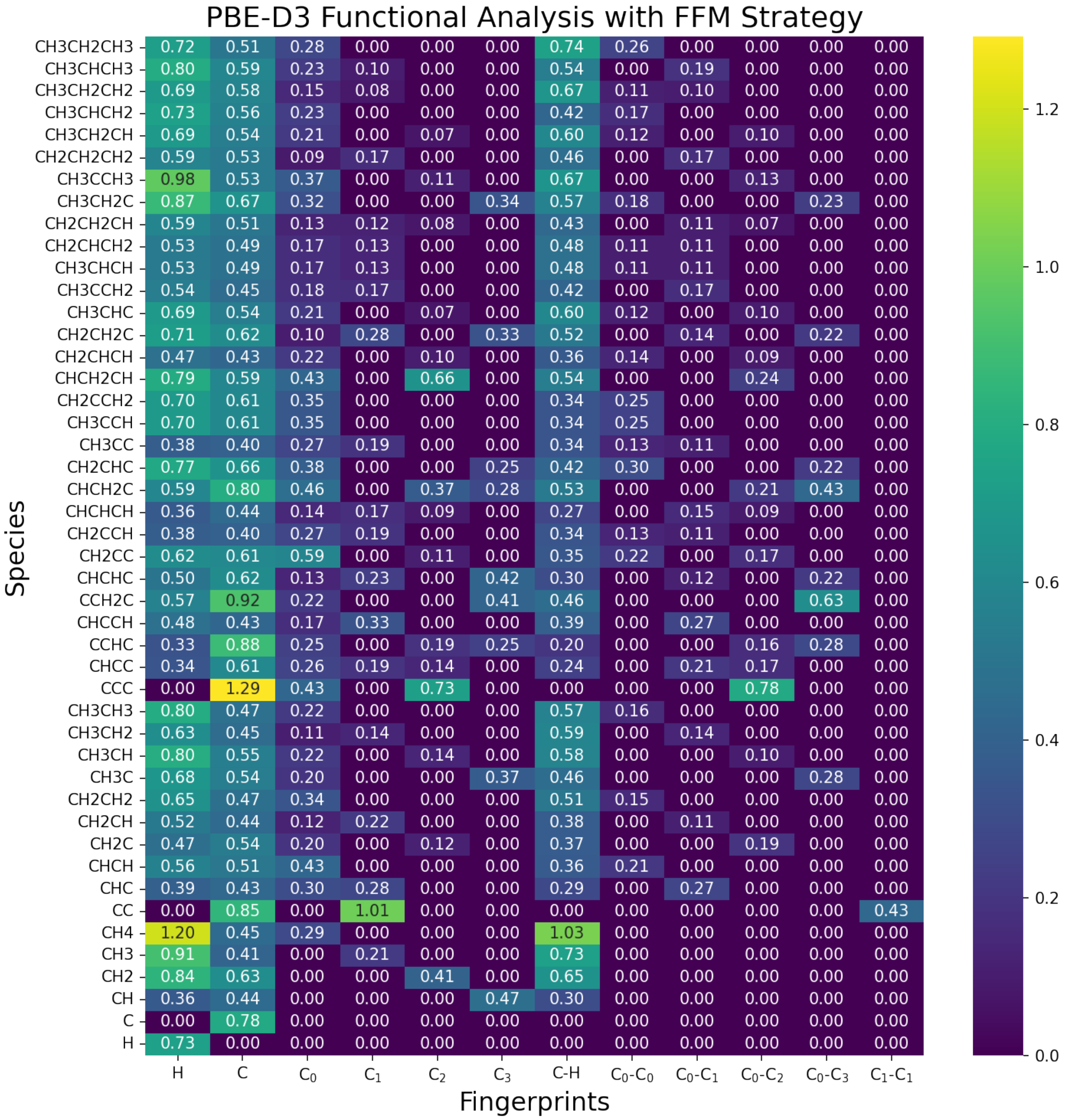

Figure S1: Species-based breakdown of fingerprint contribution for FFM training strategy and PBE-D3 functional. Each cell in the heatmap signifies the contribution of a specific fingerprint to the adsorption energy prediction for a particular molecular species. Fingerprints with negligible contributions have been omitted for clarity. The color gradient indicates the magnitude of contribution, emphasizing the impact of specific fingerprints.

The Pt(111) catalyst site model, cleaved from an optimized Pt bulk crystal, comprised of 4 layers of 4x4 atoms with a vacuum space of 20 Å between periodic slabs. Pt(111) slab and intermediate species were relaxed until the Hellmann-Feynman force[3] per atom was less than 0.03 eV Å-1. The Brillouin zone integration was sampled using a $5 \times 5 \times 1$ Monkhorst-Pack[4] k-mesh with Methfessel-Paxton smearing[5] width (σ) of 0.2 eV. Frequency calculations were performed on the optimized structures to obtain the intermediate's entropic properties and free energy. For the other functionals, single-point calculations were done to obtain the corresponding energy of the PBE-D3 optimized structures, and these VASP energies were combined with PBE-D3-based vibrational frequencies to compute their free energies.

For all four functionals, the free energies were referenced to the bare catalyst slab, gas-phase propane and gas-phase hydrogen energies.

Molecular Species Specific Fingerprint Contribution

Figure S1 illustrates an in-depth species-based fingerprint contribution breakdown employing the Four Functional Model (FFM) strategy with the PBE-D3 functional. This heatmap visualization elucidates the contributions of individual fingerprints to the prediction of adsorption energies across various molecular species. The attribution analysis presented in this heatmap has been calculated using integrated gradients, as implemented in the Python-based Captum library.[6] Through this analysis, we observe significant insights into the model's behavior; for instance, the C_2 fingerprint shows increased attribution in species such as CCC and $CHCH_2CH$, where the presence of carbon atoms with two free valencies is crucial. This analysis exemplifies how our strategy effectively capitalizes on functional invariances to learn invaluable patterns, thereby enhancing our understanding of molecular interactions within the dataset.

Table S4: Mean and standard deviation of D^2-scores for models using Original, PCA, and IMR representations derived from 24-length flat molecular fingerprints via FFM training, across 10 trials. Higher scores indicate better-fitted models.

Test Functional	ML Alg.	Original	PCA	IMR
PBE-D3	ridge	-0.01 ± 0.15	-0.02 ± 0.14	0.13 ± 0.21
	elastic	-0.07 ± 0.14	-0.09 ± 0.15	0.13 ± 0.17
	krr	-0.12 ± 0.19	-0.06 ± 0.11	0.06 ± 0.16
	svr	0.00 ± 0.16	0.04 ± 0.14	0.06 ± 0.14
BEEF-vdW	ridge	0.05 ± 0.14	0.05 ± 0.14	0.51 ± 0.08
	elastic	0.02 ± 0.11	0.00 ± 0.08	0.51 ± 0.06
	krr	-0.02 ± 0.12	-0.04 ± 0.15	0.55 ± 0.08
	svr	0.05 ± 0.18	0.05 ± 0.20	0.59 ± 0.10
RPBE	ridge	0.17 ± 0.16	0.17 ± 0.15	0.43 ± 0.14
	elastic	0.13 ± 0.10	0.12 ± 0.09	0.49 ± 0.11
	krr	0.09 ± 0.10	0.05 ± 0.14	0.42 ± 0.15
	svr	0.14 ± 0.21	0.09 ± 0.19	0.46 ± 0.12
SCAN+rVV10	ridge	0.08 ± 0.09	0.07 ± 0.08	0.40 ± 0.10
	elastic	0.01 ± 0.07	0.03 ± 0.06	0.40 ± 0.07
	krr	-0.03 ± 0.19	-0.03 ± 0.19	0.44 ± 0.09
	svr	0.05 ± 0.22	0.05 ± 0.17	0.40 ± 0.15

Goodness-of-Fit Analysis across Experimental Cases

We have utilized the D^2-score, implemented in Python's Scikit-learn[7] library, to quantify the goodness-of-fit for our models in all experimental cases. This score measures the fraction of deviance explained by the model relative to an intercept-only model and is defined as follows:

$$D^2(y, \hat{y}) = 1 - \frac{\text{dev}(y, \hat{y})}{\text{dev}(y, y_{null})} \tag{1}$$

In this equation, y represents the true target values, $\hat{y}$ denotes the predictions made by the model, and y_{null} is the median of the targets calculated on the training samples. Therefore, the term $\text{dev}(y, \hat{y})$ refers to the deviation of the model predictions from the true target values which is the mean absolute error of the model. Similarly, $\text{dev}(y, y_{null})$ signifies the mean absolute deviation of the true target values from the median calculated, serving as the baseline comparison for the model's predictive power. The best possible D^2-score is 1.0, indicating a perfect prediction. We present the mean and standard deviation of

the D^2-score calculated across all 10 trials for each experimental case scenario as in Table S4, S5, S6, S7, S8, S9, S10, S11, and S12.

Table S5: Mean and standard deviation of D^2-scores for models using Original, PCA, and IMR representations derived from 768-length chEMBL fingerprints via FFM training, across 10 trials. Higher scores indicate better-fitted models.

Test Functional	ML Alg.	Original	PCA	IMR
PBE-D3	ridge	-0.28 ± 0.22	-0.20 ± 0.17	0.19 ± 0.33
	elastic	-0.02 ± 0.13	-0.04 ± 0.13	0.24 ± 0.18
	krr	0.14 ± 0.14	0.10 ± 0.14	0.38 ± 0.18
	svr	0.12 ± 0.08	0.10 ± 0.07	0.41 ± 0.18
BEEF-vdW	ridge	-0.28 ± 0.20	-0.11 ± 0.15	0.58 ± 0.15
	elastic	-0.03 ± 0.15	0.01 ± 0.04	0.57 ± 0.16
	krr	-0.03 ± 0.21	0.02 ± 0.12	0.56 ± 0.18
	svr	0.02 ± 0.11	-0.04 ± 0.12	0.58 ± 0.16
RPBE	ridge	-0.21 ± 0.20	-0.04 ± 0.09	0.50 ± 0.18
	elastic	0.01 ± 0.15	0.01 ± 0.05	0.47 ± 0.21
	krr	-0.17 ± 0.26	0.02 ± 0.10	0.42 ± 0.14
	svr	0.00 ± 0.12	-0.04 ± 0.08	0.46 ± 0.12
SCAN+rVV10	ridge	-0.10 ± 0.18	0.03 ± 0.14	0.43 ± 0.14
	elastic	0.04 ± 0.09	0.05 ± 0.10	0.43 ± 0.15
	krr	0.08 ± 0.07	0.03 ± 0.08	0.46 ± 0.07
	svr	0.13 ± 0.10	0.01 ± 0.10	0.54 ± 0.09

Table S6: Mean and standard deviation of D^2-scores for models using Original, PCA, and IMR representations derived from 24-length Morgan fingerprints via FFM training, across 10 trials. Higher scores indicate better-fitted models.

Test Functional	ML Alg.	Original	PCA	IMR
PBE-D3	ridge	-0.08 ± 0.11	-0.07 ± 0.14	-0.08 ± 0.32
	elastic	-0.01 ± 0.06	-0.01 ± 0.08	0.19 ± 0.13
	krr	-0.01 ± 0.10	-0.04 ± 0.15	0.10 ± 0.11
	svr	-0.01 ± 0.11	0.01 ± 0.08	0.11 ± 0.14
BEEF-vdW	ridge	-0.03 ± 0.10	-0.02 ± 0.10	0.66 ± 0.08
	elastic	0.01 ± 0.04	-0.02 ± 0.05	0.66 ± 0.08
	krr	-0.01 ± 0.06	-0.02 ± 0.06	0.69 ± 0.06
	svr	0.01 ± 0.11	0.02 ± 0.11	0.70 ± 0.06
RPBE	ridge	0.02 ± 0.09	0.03 ± 0.09	0.53 ± 0.09
	elastic	0.02 ± 0.03	-0.01 ± 0.03	0.56 ± 0.07
	krr	0.00 ± 0.06	-0.03 ± 0.06	0.54 ± 0.08
	svr	0.01 ± 0.08	0.00 ± 0.07	0.56 ± 0.11
SCAN+rVV10	ridge	0.03 ± 0.14	0.04 ± 0.14	0.45 ± 0.12
	elastic	0.01 ± 0.06	0.03 ± 0.06	0.52 ± 0.11
	krr	0.04 ± 0.06	0.04 ± 0.07	0.57 ± 0.04
	svr	0.03 ± 0.06	0.05 ± 0.07	0.54 ± 0.06

Table S7: Mean and standard deviation of D^2-scores for models using Original, PCA, and IMR representations derived from 24-length flat molecular fingerprints via BEM training, across 10 trials. Higher scores indicate better-fitted models.

Test Functional	ML Alg.	Original	PCA	IMR
PBE-D3	ridge	-0.01 ± 0.15	-0.02 ± 0.14	0.21 ± 0.11
	elastic	-0.07 ± 0.14	-0.10 ± 0.15	0.18 ± 0.08
	krr	-0.12 ± 0.19	-0.06 ± 0.11	0.25 ± 0.14
	svr	0.00 ± 0.16	0.04 ± 0.14	0.22 ± 0.12
BEEF-vdW	ridge	0.05 ± 0.14	0.05 ± 0.14	0.42 ± 0.11
	elastic	0.02 ± 0.11	0.00 ± 0.09	0.42 ± 0.12
	krr	-0.02 ± 0.12	-0.06 ± 0.15	0.59 ± 0.11
	svr	0.05 ± 0.18	0.05 ± 0.20	0.58 ± 0.12
RPBE	ridge	0.17 ± 0.16	0.17 ± 0.16	0.33 ± 0.18
	elastic	0.13 ± 0.10	0.13 ± 0.09	0.39 ± 0.15
	krr	0.09 ± 0.10	0.04 ± 0.13	0.50 ± 0.09
	svr	0.14 ± 0.21	0.09 ± 0.19	0.46 ± 0.13
SCAN+rVV10	ridge	0.08 ± 0.09	0.07 ± 0.08	0.42 ± 0.10
	elastic	0.01 ± 0.07	0.03 ± 0.06	0.43 ± 0.11
	krr	-0.03 ± 0.19	-0.03 ± 0.19	0.51 ± 0.07
	svr	0.05 ± 0.22	0.05 ± 0.17	0.47 ± 0.09

Table S8: Mean and standard deviation of D^2-scores for models using Original, PCA, and IMR representations derived from 768-length chEMBL fingerprints via BEM training, across 10 trials. Higher scores indicate better-fitted models.

Test Functional	ML Alg.	Original	PCA	IMR
PBE-D3	ridge	-0.28 ± 0.22	-0.16 ± 0.21	0.18 ± 0.10
	elastic	-0.02 ± 0.13	0.00 ± 0.10	0.29 ± 0.12
	krr	0.14 ± 0.14	0.09 ± 0.09	0.21 ± 0.14
	svr	0.12 ± 0.08	0.06 ± 0.11	0.14 ± 0.18
BEEF-vdW	ridge	-0.28 ± 0.20	-0.13 ± 0.16	0.60 ± 0.12
	elastic	-0.03 ± 0.15	-0.01 ± 0.04	0.59 ± 0.14
	krr	-0.03 ± 0.21	-0.01 ± 0.12	0.67 ± 0.12
	svr	0.02 ± 0.11	-0.03 ± 0.12	0.56 ± 0.18
RPBE	ridge	-0.21 ± 0.20	-0.01 ± 0.10	0.55 ± 0.09
	elastic	0.01 ± 0.15	0.00 ± 0.04	0.57 ± 0.06
	krr	-0.17 ± 0.26	0.02 ± 0.10	0.54 ± 0.09
	svr	0.00 ± 0.12	-0.03 ± 0.08	0.58 ± 0.14
SCAN+rVV10	ridge	-0.10 ± 0.18	0.05 ± 0.13	0.40 ± 0.23
	elastic	0.04 ± 0.09	0.03 ± 0.09	0.43 ± 0.13
	krr	0.08 ± 0.07	0.04 ± 0.09	0.57 ± 0.09
	svr	0.13 ± 0.10	0.03 ± 0.11	0.52 ± 0.08

Table S9: Mean and standard deviation of D^2-scores for models using Original, PCA, and IMR representations derived from 24-length Morgan fingerprints via BEM training, across 10 trials. Higher scores indicate better-fitted models.

Test Functional	ML Alg.	Original	PCA	IMR
PBE-D3	ridge	-0.08 ± 0.11	-0.06 ± 0.12	0.01 ± 0.19
	elastic	-0.01 ± 0.06	-0.01 ± 0.08	0.28 ± 0.12
	krr	-0.01 ± 0.10	-0.08 ± 0.19	0.15 ± 0.13
	svr	-0.01 ± 0.11	-0.03 ± 0.15	0.20 ± 0.13
BEEF-vdW	ridge	-0.03 ± 0.10	-0.03 ± 0.09	0.66 ± 0.05
	elastic	0.01 ± 0.04	0.00 ± 0.06	0.64 ± 0.08
	krr	-0.01 ± 0.06	-0.03 ± 0.07	0.71 ± 0.13
	svr	0.01 ± 0.11	0.00 ± 0.11	0.70 ± 0.15
RPBE	ridge	0.02 ± 0.09	0.02 ± 0.10	0.52 ± 0.20
	elastic	0.02 ± 0.03	-0.02 ± 0.04	0.49 ± 0.34
	krr	0.00 ± 0.06	0.00 ± 0.06	0.52 ± 0.17
	svr	0.01 ± 0.08	0.01 ± 0.07	0.52 ± 0.10
SCAN+rVV10	ridge	0.03 ± 0.14	0.03 ± 0.13	0.44 ± 0.13
	elastic	0.01 ± 0.06	0.03 ± 0.07	0.45 ± 0.14
	krr	0.04 ± 0.06	0.03 ± 0.07	0.53 ± 0.06
	svr	0.03 ± 0.06	0.03 ± 0.06	0.45 ± 0.13

Table S10: Mean and standard deviation of D^2-scores for models using Original, PCA, and IMR representations derived from 24-length flat molecular fingerprints via FSM training, across 10 trials. Higher scores indicate better-fitted models.

Test Functional	ML Alg.	Original	PCA	IMR
PBE-D3	ridge	-0.01 ± 0.15	-0.02 ± 0.14	-0.08 ± 0.14
	elastic	-0.07 ± 0.14	-0.09 ± 0.15	-0.07 ± 0.14
	krr	-0.12 ± 0.19	-0.05 ± 0.10	-0.22 ± 0.25
	svr	0.00 ± 0.16	0.04 ± 0.14	-0.13 ± 0.15
BEEF-vdW	ridge	0.05 ± 0.14	0.05 ± 0.14	0.04 ± 0.11
	elastic	0.02 ± 0.11	0.01 ± 0.10	0.02 ± 0.11
	krr	-0.02 ± 0.12	-0.06 ± 0.16	-0.11 ± 0.27
	svr	0.05 ± 0.18	0.05 ± 0.20	-0.11 ± 0.27
RPBE	ridge	0.17 ± 0.16	0.17 ± 0.15	0.15 ± 0.15
	elastic	0.13 ± 0.10	0.13 ± 0.09	0.14 ± 0.12
	krr	0.09 ± 0.10	0.04 ± 0.14	0.03 ± 0.28
	svr	0.14 ± 0.21	0.09 ± 0.19	0.09 ± 0.27
SCAN+rVV10	ridge	0.08 ± 0.09	0.07 ± 0.08	0.07 ± 0.07
	elastic	0.01 ± 0.07	0.03 ± 0.06	0.04 ± 0.05
	krr	-0.03 ± 0.19	-0.03 ± 0.19	0.00 ± 0.25
	svr	0.05 ± 0.22	0.05 ± 0.17	-0.02 ± 0.24

Table S11: Mean and standard deviation of D^2-scores for models using Original, PCA, and IMR representations derived from 768-length chEMBL fingerprints via FSM training, across 10 trials. Higher scores indicate better-fitted models.

Test Functional	ML Alg.	Original	PCA	IMR
PBE-D3	ridge	-0.28 ± 0.22	-0.15 ± 0.16	0.00 ± 0.11
	elastic	-0.02 ± 0.13	-0.06 ± 0.14	0.00 ± 0.12
	krr	0.14 ± 0.14	0.10 ± 0.06	0.02 ± 0.09
	svr	0.12 ± 0.08	0.09 ± 0.07	0.00 ± 0.09
BEEF-vdW	ridge	-0.28 ± 0.20	-0.12 ± 0.14	-0.03 ± 0.12
	elastic	-0.03 ± 0.15	0.00 ± 0.03	-0.02 ± 0.12
	krr	-0.03 ± 0.21	0.02 ± 0.12	-0.04 ± 0.13
	svr	0.02 ± 0.11	-0.04 ± 0.11	-0.04 ± 0.14
RPBE	ridge	-0.21 ± 0.20	-0.03 ± 0.10	0.00 ± 0.12
	elastic	0.01 ± 0.15	0.02 ± 0.05	0.01 ± 0.12
	krr	-0.17 ± 0.26	0.02 ± 0.10	0.00 ± 0.16
	svr	0.00 ± 0.12	-0.05 ± 0.07	-0.01 ± 0.15
SCAN+rVV10	ridge	-0.10 ± 0.18	0.05 ± 0.13	0.10 ± 0.16
	elastic	0.04 ± 0.09	0.04 ± 0.10	0.11 ± 0.15
	krr	0.08 ± 0.07	0.04 ± 0.09	0.13 ± 0.14
	svr	0.13 ± 0.10	0.03 ± 0.10	0.12 ± 0.15

Table S12: Mean and standard deviation of D^2-scores for models using Original, PCA, and IMR representations derived from 24-length Morgan fingerprints via FSM training, across 10 trials. Higher scores indicate better-fitted models.

Test Functional	ML Alg.	Original	PCA	IMR
PBE-D3	ridge	-0.08 $\pm$ 0.11	-0.08 $\pm$ 0.13	-0.15 $\pm$ 0.16
	elastic	-0.01 $\pm$ 0.06	0.00 $\pm$ 0.04	-0.10 $\pm$ 0.22
	krr	-0.01 $\pm$ 0.10	-0.02 $\pm$ 0.11	-0.21 $\pm$ 0.39
	svr	-0.01 $\pm$ 0.11	0.01 $\pm$ 0.08	-0.26 $\pm$ 0.39
BEEF-vdW	ridge	-0.03 $\pm$ 0.10	-0.03 $\pm$ 0.10	-0.02 $\pm$ 0.13
	elastic	0.01 $\pm$ 0.04	0.00 $\pm$ 0.06	-0.04 $\pm$ 0.18
	krr	-0.01 $\pm$ 0.06	-0.02 $\pm$ 0.08	-0.04 $\pm$ 0.16
	svr	0.01 $\pm$ 0.11	0.01 $\pm$ 0.12	-0.02 $\pm$ 0.16
RPBE	ridge	0.02 $\pm$ 0.09	0.02 $\pm$ 0.09	-0.01 $\pm$ 0.11
	elastic	0.02 $\pm$ 0.03	-0.02 $\pm$ 0.03	-0.03 $\pm$ 0.12
	krr	0.00 $\pm$ 0.06	-0.02 $\pm$ 0.08	-0.12 $\pm$ 0.16
	svr	0.01 $\pm$ 0.08	0.01 $\pm$ 0.07	-0.04 $\pm$ 0.22
SCAN+rVV10	ridge	0.03 $\pm$ 0.14	0.03 $\pm$ 0.13	0.00 $\pm$ 0.19
	elastic	0.01 $\pm$ 0.06	0.03 $\pm$ 0.07	-0.02 $\pm$ 0.14
	krr	0.04 $\pm$ 0.06	0.03 $\pm$ 0.07	-0.01 $\pm$ 0.15
	svr	0.03 $\pm$ 0.06	0.03 $\pm$ 0.06	0.00 $\pm$ 0.15

References

(1) Perdew, J. P.; Burke, K.; Ernzerhof, M. Generalized gradient approximation made simple. *Phys. Rev. Lett.* **1996**, *77*, 3865.

(2) Grimme, S.; Antony, J.; Ehrlich, S.; Krieg, H. A consistent and accurate ab initio parametrization of density functional dispersion correction (DFT-D) for the 94 elements H-Pu. *J. Chem. Phys.* **2010**, *132*, 154104.

(3) Di Ventra, M.; Pantelides, S. T. Hellmann-Feynman theorem and the definition of forces in quantum time-dependent and transport problems. *Phys. Rev. B* **2000**, *61*, 16207.

(4) Monkhorst, H. J.; Pack, J. D. Special points for Brillouin-zone integrations. *Phys. Rev. B* **1976**, *13*, 5188.

(5) Methfessel, M.; Paxton, A. High-precision sampling for Brillouin-zone integration in metals. *Phys. Rev. B* **1989**, *40*, 3616.

(6) Kokhlikyan, N.; Miglani, V.; Martin, M.; Wang, E.; Alsallakh, B.; Reynolds, J.; Melnikov, A.; Kliushkina, N.; Araya, C.; Yan, S.; Reblitz-Richardson, O. Captum: A unified and generic model interpretability library for Pytorch. *arXiv preprint arXiv:2009.07896* **2020**,

(7) Pedregosa, F.; Varoquaux, G.; Gramfort, A.; Michel, V.; Thirion, B.; Grisel, O.; Blondel, M.; Prettenhofer, P.; Weiss, R.; Dubourg, V.; Vanderplas, J. Scikit-learn: Machine Learning in Python. *J. Mach. Learn. Res.* **2011**, *12*, 2825–2830.
